# Health record hiccups—5,526 real-world time series with change points labelled by crowdsourced visual inspection

**DOI:** 10.1093/gigascience/giad060

**Published:** 2023-07-28

**Authors:** T Phuong Quan, Ben Lacey, Tim E A Peto, A Sarah Walker

**Affiliations:** Nuffield Department of Clinical Medicine, University of Oxford, Oxford OX3 9DU, UK; Nuffield Department of Population Health, University of Oxford, Oxford OX3 7LF, UK; Nuffield Department of Clinical Medicine, University of Oxford, Oxford OX3 9DU, UK; Nuffield Department of Clinical Medicine, University of Oxford, Oxford OX3 9DU, UK

**Keywords:** time series, change point detection, anomalies, data quality

## Abstract

**Background:**

Large routinely collected data such as electronic health records (EHRs) are increasingly used in research, but the statistical methods and processes used to check such data for temporal data quality issues have not moved beyond manual, ad hoc production and visual inspection of graphs. With the prospect of EHR data being used for disease surveillance via automated pipelines and public-facing dashboards, automation of data quality checks will become increasingly valuable.

**Findings:**

We generated 5,526 time series from 8 different EHR datasets and engaged >2,000 citizen-science volunteers to label the locations of all suspicious-looking change points in the resulting graphs. Consensus labels were produced using density-based clustering with noise, with validation conducted using 956 images containing labels produced by an experienced data scientist. Parameter tuning was done against 670 images and performance calculated against 286 images, resulting in a final sensitivity of 80.4% (95% CI, 77.1%–83.3%), specificity of 99.8% (99.7%–99.8%), positive predictive value of 84.5% (81.4%–87.2%), and negative predictive value of 99.7% (99.6%–99.7%). In total, 12,745 change points were found within 3,687 of the time series.

**Conclusions:**

This large collection of labelled EHR time series can be used to validate automated methods for change point detection in real-world settings, encouraging the development of methods that can successfully be applied in practice. It is particularly valuable since change point detection methods are typically validated using synthetic data, so their performance in real-world settings cannot be assumed to be comparable. While the dataset focusses on EHRs and data quality, it should also be applicable in other fields.

## Data Description

### Context

The use of electronic health records (EHRs) in medical research has grown enormously over the past 20 years, given its ability to cover large numbers of patients and often over long time periods. However, using routinely collected data such as EHRs for research carries inherent risks, since the data will have been collected for a different purpose (i.e., operational) and usually at a great distance (both temporally and physically) from the researchers making use of it. Therefore, to ensure the validity of their research outputs, it is important that researchers include checks for data quality issues before conducting their analyses [1].

**Figure 1:**
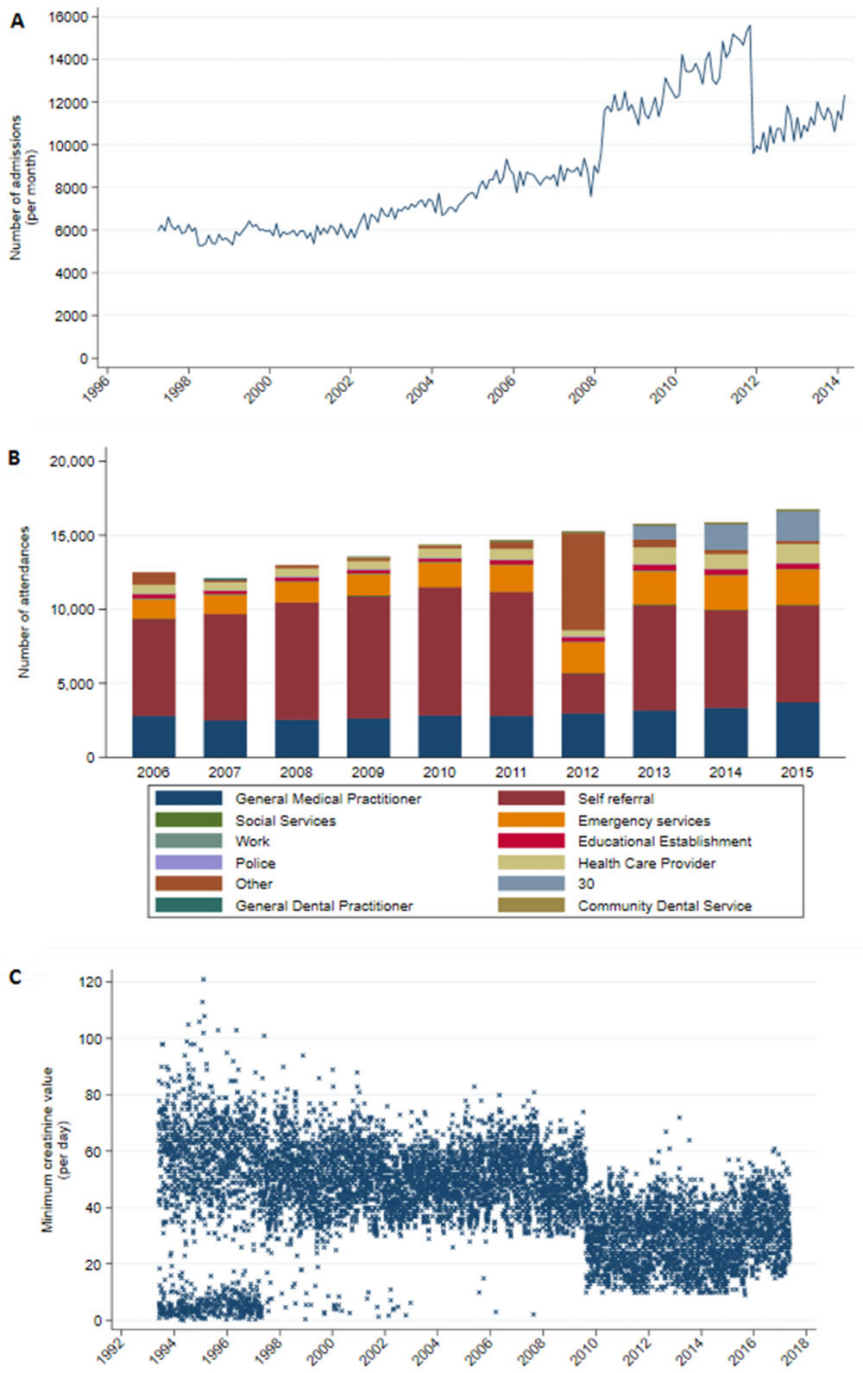
Examples of temporal changes in data caused by updates to infrastructure at Oxford University Hospitals. (A) Total number of inpatient admissions containing multiple diagnosis codes. The jump in records in 2008 was caused by the inclusion of dialysis day-case patients, which were then excluded again in 2012. (B) Emergency department attendances by referral source. A change in computer systems in 2011 noticeably affected the data recorded, with the “Other” category temporarily being overrepresented in 2012, and a new, undefined category of “30” appearing thereafter. (C) Lowest creatinine blood test result each day. The bimodal distribution up to 1997 was due to a mixture of units being used, and the drop in values in 2009 was due to a change in testing method and reference range.

In particular, the presence of change points (i.e., points in time where the distribution of data values changes suddenly and unpredictably) can lead to systematic biases that, if not identified and taken into account, can lead to erroneous results and incorrect conclusions being drawn, ultimately resulting in poor decisions at a clinical or public health policy level. For example, Fig. 1 shows 3 real-world examples of data from a large UK hospital group and where shifts in the data were caused by changes to infrastructure rather than by natural changes in the patient population. If a researcher were to naively compare the number of hospital admissions (Fig. 1A) in 2010 to the number of admissions in 2013, without checking for change points in between those dates, they could incorrectly conclude that hospital admissions had decreased when in fact they had been increasing. Similarly, a researcher analysing a cohort of patients between 2008 and 2012 might mistakenly infer that the patients admitted with infections in 2012 were overall less severely unwell than those admitted in 2008 because they had lower creatinine blood test values (Fig. 1C), when in fact the difference was due to a change in testing method and not in the patients themselves.

While these types of temporal artefacts should in theory be picked up by the diligent researcher at the initial data analysis stage, in practice, it is not clear to what extent this is actually done, since this process is rarely, if ever, reported in published papers [2, 3]. Standard checks such as the calculation of summary statistics and visual inspection of graphs may be effective enough for traditional research studies where there is a limited number of variables of interest as well as a researcher with appropriate domain knowledge, but with the increasing volume of data being collected in EHRs and across multiple sites (each with their own idiosyncratic processes), these checks will become more and more onerous and therefore less likely to be conducted thoroughly and consistently. Therefore, automation of checks that would otherwise be labour intensive and repetitive, such as screening time series for change points, would be of value to researchers. Furthermore, there is an increasing prospect of EHR data being used for disease surveillance via automated pipelines and public-facing dashboards, where automation of data quality checks will be of even more value.

While there is a rich literature on change point detection methods, with applications across a range of different scientific fields [4], none of these has to our knowledge been developed with a focus on EHRs or on data quality. Additionally, most of these methods are validated using synthetic data, and as such, their advertised performance cannot be assumed to hold in real-world scenarios. Therefore, in order to assess whether or not any of these methods would be effective to use as a screening method for identifying change points in EHRs requires real-world datasets with “gold-standard” labels against which to judge performance.

### Methods

**Figure 2:**
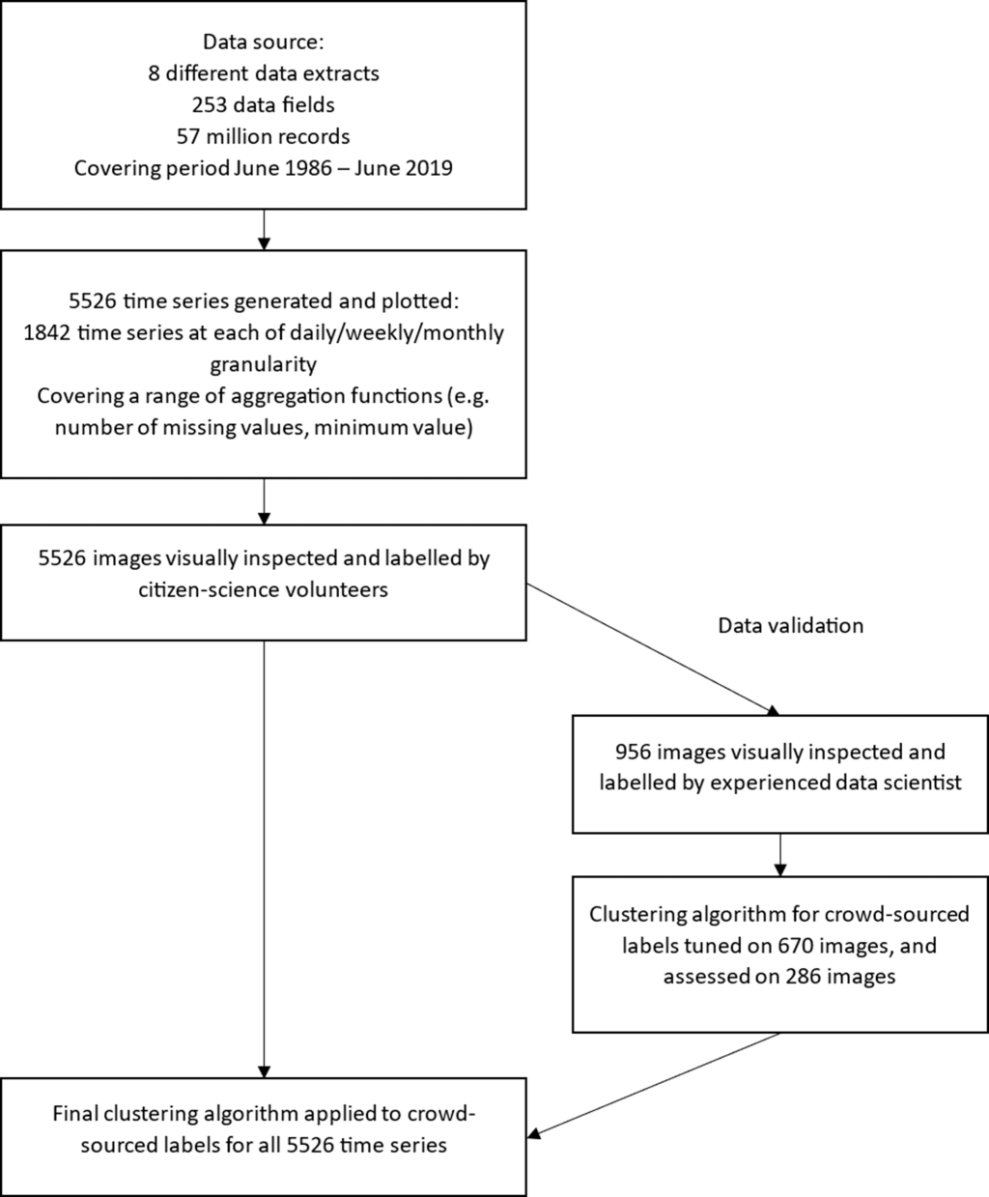
Overview of the dataset creation workflow.

An overview of the process can be seen in Fig. 2, with full details described below.

#### Study sample

EHR data are collected from all patients attending the 4 hospitals within the Oxford University Hospitals NHS Foundation Trust (OUH), which provide all acute care and all microbiology and pathology services in the region (∼600,000 individuals). Much of these data is automatically fed into a linked database for use in surveillance and service activities within the OUH and is periodically extracted into a partially curated, anonymised, research database, the Infections in Oxfordshire Research Database (IORD). These data go back to the 1980s and are known to cover multiple periods of change in the hospital computer and laboratory systems.

IORD has Research Ethics Committee and Health Research Authority approval as a generic deidentified electronic research database (19/SC/0403, 19/CAG/0144).

Data were included from all 4 major component datasets of IORD (patient administration, antibiotic prescribing, haematology/biochemistry laboratories, and microbiology laboratories). Eight data extracts were taken, comprising a total of 253 data fields and 57 million records, with dates between 2 June 1986 and 30 June 2019:

- Inpatient episodes
- Outpatient episodes
- Emergency department episodes
- Antibiotic prescriptions
- Biochemistry creatinine tests (a common biomarker for infection)
- Haematology neutrophil counts (a standard test requested for most patients)
- Microbiology blood culture tests
- Microbiology tests that identified *Esherichia coli* (regardless of specimen type)

#### Creation of time series

A total of 5,526 time series were generated from the 8 data extracts, as follows.

One data field from each data extract was selected to be its “**timepoint**” field, and this was used to represent the date of the record (patient administration data used the discharge date, laboratory data used the specimen collection date, and antibiotic data used the prescription date). Any records that contained a missing or invalid datetime value in the timepoint field were necessarily excluded. Also, any duplicate records were removed, and the number of removed records stored as a calculated field.

##### Aggregation granularities

For each data extract, the time span that each timepoint field covered was divided into regular intervals. Records were aggregated using the chosen timepoint field by **day** (midnight to midnight), as well as by **week** (Monday to Sunday) and by calendar **month**.

##### Aggregation functions

Numeric summary values were calculated for each timepoint from the (often nonnumeric) data by applying simple functions (e.g., number of values present, percentage of missing values, number of distinct values, or median value). If there were no records in a particular timepoint (which meant that no summary value could be calculated), the value of NA was given (except for the aggregation function counting the number of values present in a data field, which would take the value of 0 as expected). Each aggregation function demonstrated a measure within one of the intrinsic data quality dimensions of Completeness, Conformance, and Plausibility [5]. Different functions were used depending on the type of data field:

- **Timepoint—**The data field representing the date of the event described in the record
- **Numeric—**Fields containing continuous values (such as blood cell counts) or discrete integers (such as the episode number within an admission spell)
- **Categorical—**Fields containing a finite list of values, which may be stored either as character strings or coded as integers
- **Datetime—**Fields containing dates, with or without a time element
- **UniqueIdentifier—**Fields containing computer-generated record identifiers and may be based on either a numeric or a character data type
- **Freetext—**Unstructured text

or if applied to the data extract as a whole (e.g., calculating the number of duplicate records). See Table 1 for details of the data fields in each data extract and Table 2 for the list of aggregation functions applied to each data field.

**Table 1:** Overview of data fields contained in each data extract

Dataset type	Data extract	Data from	Data to	Total No. of data fields^a^	No. of timepoint fields	No. of numeric fields	No. of categorical fields	No. of datetime fields	No. of UniqueIdentifier fields	No. of freetext fields
Antibiotics	Antibiotic prescribing	10/06/2008	30/06/2019	27	1	3	9	7	2	3
Patient administration	Emergency department attendances	01/04/2005	30/06/2019	28	1	1	15	6	2	1
Patient administration	Inpatient episodes	01/04/1997	30/06/2019	41	1	2	23	6	4	3
Patient administration	Outpatient episodes	01/04/1997	30/06/2019	35	1	1	21	4	3	3
Biochemistry	Creatinine tests	02/06/1986	30/06/2019	24	1	1	7	5	6	2
Haematology	Neutrophil counts	01/04/1987	30/06/2019	24	1	1	7	5	6	2
Microbiology	Blood cultures	04/06/1993	30/06/2019	37	1	0	18	6	2	8
Microbiology	*E. coli* isolations	17/05/1993	30/06/2019	37	1	0	18	6	2	8

a Includes 2calculated fields, for duplicate records and for all data combined.

**Table 2:** The aggregation functions applied to each data field, to produce the time series

	Individual data field type						Across data extract as a whole	
Aggregation function (*shorthand label*)	Timepoint	Numeric	Categorical	Datetime	UniqueIdentifier	Freetext	All data combined	Duplicate records
**COMPLETENESS**								
Number of missing values (*missing_n*)		x	x	x	x	x	x	
Percentage of missing values (*missing_perc*)		x	x	x	x	x	x	
**CONFORMANCE**								
Number of nonconformant values^a^ (*nonconformant_n*)		x		x			x	
Percentage of nonconformant values^a^ (*nonconformant_perc*)		x		x			x	
**PLAUSIBILITY**								
Sum of duplicate records removed (*sum*)								x
Percentage of records that had been duplicated (*nonzero_perc*)								x
Number of values present (*n*)	x	x	x	x	x	x	x	
Minimum value (*min*)		x		x				
Maximum value (*max*)		x		x				
Mean value (*mean*)		x						
Median value (*median*)		x						
Number of values with no time element^b^ (*midnight_n*)	x			x				
Percentage of values with no time element^b^ (*midnight_perc*)	x			x				
Minimum string length (*minlength*)					x			
Maximum string length (*maxlength*)					x			
Mean string length (*meanlength*)					x			
Number of distinct values (*distinct*)			x					
Number of values within each subcategory^c^ (*subcat_n*)			x					
Percentage of values within each subcategory^c^ (*subcat_perc*)			x					

a Nonconformance was deemed as a nonnumeric value in a (supposedly) numeric data field or a nondate value in a (supposedly) date field.

b These were only calculated for fields that were known to contain a time element and where midnight would be used as the default when no time element was available.

c With 1 time series created per subcategory. These were only calculated for fields with fewer than 20 subcategories (with the additional inclusion of DischargeDestinationCode in the inpat_episode data extract, which contained 23 subcategories, and was included for consistency with the other coded fields in the data extract).

#### Collection of change point labels by visual inspection

**Figure 3:**
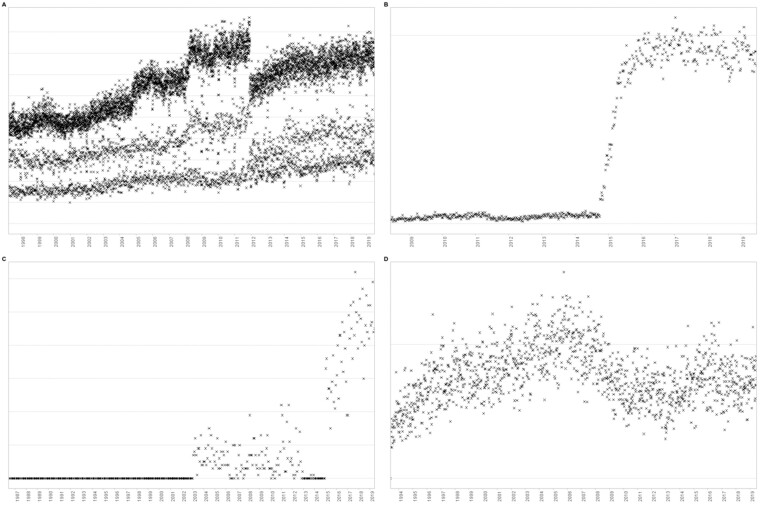
Examples of graphs generated for visual inspection of change points.

Each time series was plotted on a separate graph (with time on the x-axis and the aggregation function value on the y-axis); see Fig. 3 for some examples. Frequency-based aggregation functions were plotted on a scale always starting at zero and ending no earlier than 10. Percentages were always plotted on a 0–100 scale, and frequencies of subcategories were plotted on the same scale as frequencies for the data field as a whole. All graphs were saved as png files of the same size (i.e., 1,000px wide by 666px tall) at a resolution of 96 dpi.

Visually inspected labels for the locations of change points were collected using the Zooniverse [6] citizen-science platform. The Zooniverse is a free, popular, and well-established online platform for public involvement in research and has over 2 million registered volunteers who review and participate in multiple projects from astronomy to wildlife surveys to historical transcriptions.

**Figure 4:**
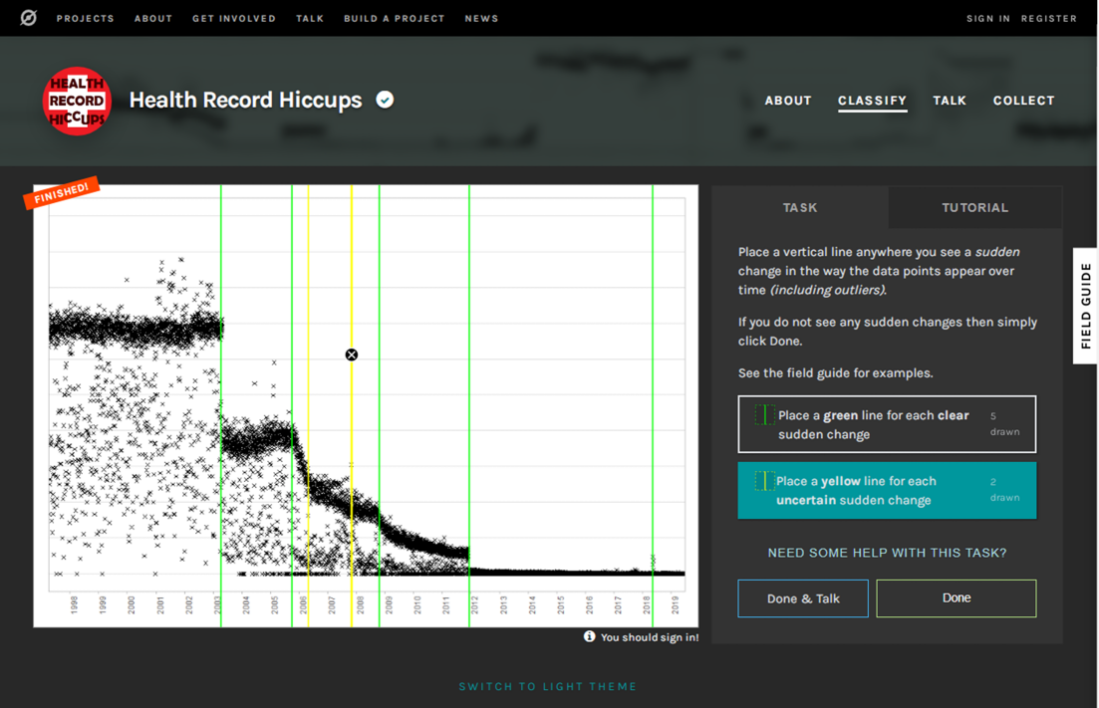
Screenshot of Zooniverse project interface.

The “Health Record Hiccups” [7] Zooniverse project showed volunteers one image at a time and asked them to draw a vertical line on the image wherever they saw an abrupt change in the distribution of values; see Fig. 4 for a screenshot. They were initially presented with a tutorial that included multiple examples of different ways in which the data can change—namely, changes in **level, trend**, (vertical) **variability, presence/absence** of data points, or (unpredictable) **outliers**. They were asked to draw a green line if they saw a clear change, a yellow line if they were uncertain, or no lines if they saw no abrupt changes. To reduce risk of bias, no metadata were visible at the point of classification.

Images were scheduled for retirement once 41 classifications had been completed on them (i.e., once the image had been inspected by 41 different people).

#### Data cleaning

Due to the way the Zooniverse platform randomises and supplies images to its volunteers, it was possible for the same person to be served the same image more than once and for images to have more than the specified number of 41 classifications. Therefore, only the first attempt per person per image was kept, up to a maximum of 41 different people per image.

**Figure 5:**
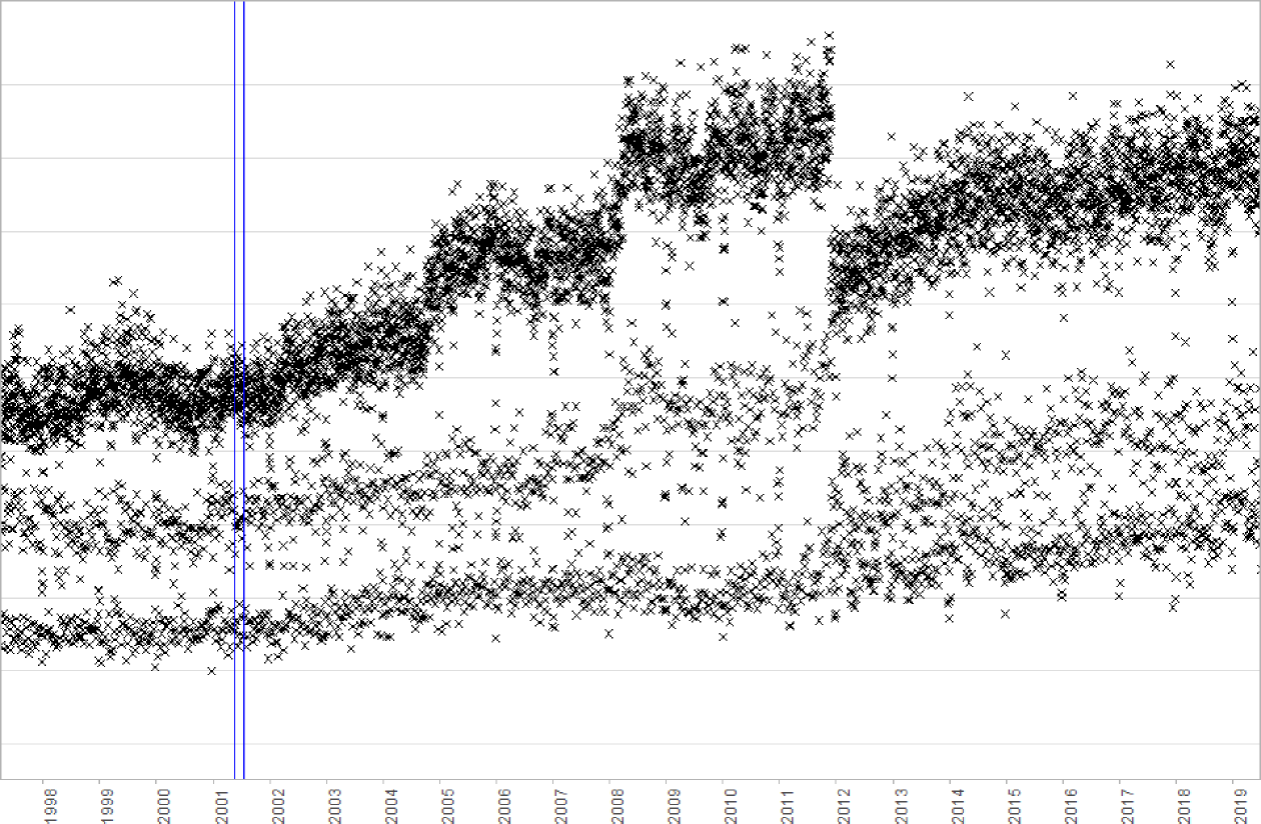
Example of 2 lines drawn 7px apart. Any lines drawn closer together than this were considered to represent the same change point.

To improve consistency between classifications made by different volunteers using different screen resolutions, a “minimum distance cutoff” of 7px was selected (see Data validation section) to distinguish between distinct change points (i.e., any lines drawn closer together than this should be assumed to represent the same change point). An example of a 7px distance between 2 lines is shown in Fig. 5. Any lines that were drawn by the same person within this “minimum distance cutoff” interval were combined into a single line located at the mean position of the contributing lines. If any of the combined lines was green (certain), the resulting line was also considered green.

#### Creation of consensus labels

To create consensus labels from the volunteers’ classifications, the *dbscan* [8] (density-based spatial clustering of applications with noise [9]) package (v1.1–5) in R (v3.6.3) was used to find zero or more clusters of lines within an image. The mean cluster location was assigned to be the crowdsourced consensus label for the change point, and any lines that were deemed by the package to be noise were ignored. Following tuning of the *dbscan* algorithm (see Data validation section), the following 3 parameters were used to create the final labels for the locations of change points:

- exclude yellow (uncertain) lines,
- minimum-lines-in-cluster (i.e., the minimum number of lines needed to create a cluster) = 5,
- epsilon-neighbourhood (i.e., the maximum distance between 2 lines in a cluster) = 3px.

**Figure 6:**
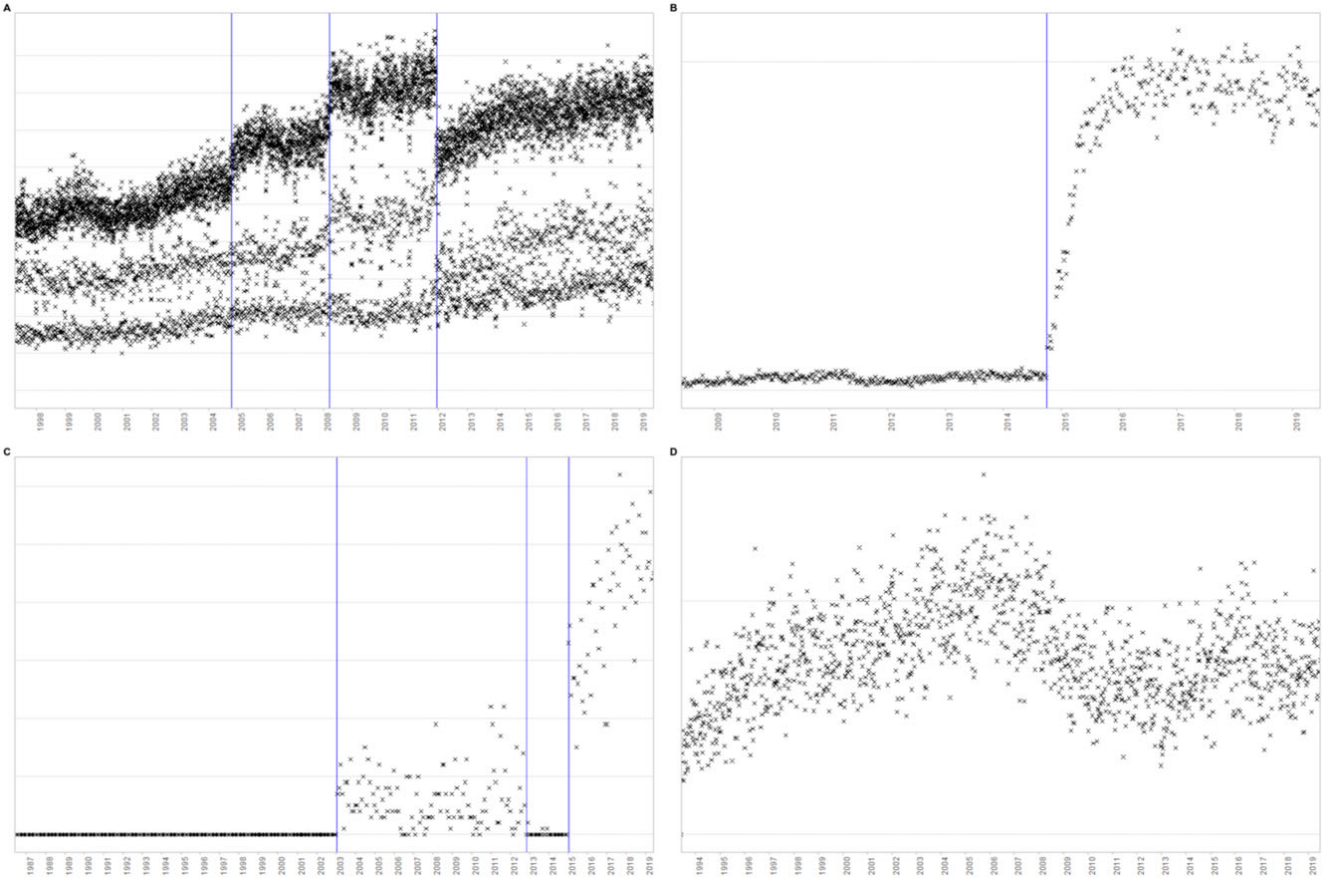
Examples of the locations of crowdsourced consensus labels for change points.

The pixel locations of the consensus labels were then converted back to dates. A total of 12,745 change points were found within 3,687 of the time series. Examples of the locations of crowdsourced consensus labels can be seen in Fig. 6. A summary of the number of change points and time series per data extract is shown in Table 3.

**Table 3:** Overview of time series and change points per data extract.

Data extract	No. of time series created	No. of constant time series (%)	No. with missing values (%)	No. with at least 1 change point (%)	Total No. of change points
**Antibiotic prescribing**	501	92 (18)	167 (33)	385 (77)	932
**Emergency department attendances**	762	87 (11)	454 (60)	528 (69)	1,589
**Inpatient episodes**	1203	129 (11)	0 (0)	665 (55)	2,064
**Outpatient episodes**	690	52 (8)	14 (2)	546 (79)	1,959
**Creatinine tests**	552	79 (14)	338 (61)	415 (75)	2,017
**Neutrophil counts**	462	83 (18)	34 (7)	356 (77)	1,584
**Blood cultures**	612	94 (15)	177 (29)	307 (50)	844
***E. coli* isolations**	744	130 (17)	218 (29)	485 (65)	1,756
**Total**	5,526	746 (13)	1,402 (25)	3,687 (67)	12,745

## Data Validation

### Methods

Accuracy of the crowdsourced consensus labels was assessed against expert labels produced for the initial batch of 956 images (inpatient episodes, antibiotic prescriptions, creatinine tests, and blood culture tests, aggregated by day). These expert labels were created by a researcher with >8 years’ experience compiling and analysing EHR data, and this was done using the same interface as the volunteers but blinded to any of their results.

To improve consistency between classifications made by different volunteers using different screen resolutions, a “minimum distance cutoff” was selected to distinguish between distinct change points (i.e., any lines drawn closer together than this should be assumed to represent the same change point). This was done by calculating the minimum distance between any 2 lines drawn on a single image by the same volunteer and the distribution of minimum distances visually inspected for a threshold.

To calculate the accuracy of the consensus crowdsourced labels compared to expert labels, a binary classifier was approximated using the following terms:

- **True positive**: a crowdsourced label is within the “minimum distance cutoff” of an expert line.
- **False positive**: a crowdsourced label is present, but no expert line lies within the “minimum distance cutoff” of it.
- **False negative**: an expert line is present, but no crowdsourced label lies within the “minimum distance cutoff” of it.
- **True negative**: total estimated as the number of the “minimum distance cutoff” intervals in an image (i.e., the maximum number of change points that could possibly be identified on a single image) minus the sum of above 3 categories.

In order to avoid double-counting, the following additional rules were enforced:

- When there were “x” crowdsourced labels close to 1 expert line, this counted as 1 true positive and zero false positives.
- When there were 2 expert lines close to 1 crowdsourced label, this counted as 2 true positives and zero false negatives.

Tuning of the algorithm to create consensus labels from the crowdsourced data was done using a random sample of 70% of the 956 images, balanced across the 4 data extracts, with the remaining 30% reserved for final testing of the performance of the algorithm. Final performance was assessed using sensitivity, specificity, positive predictive value (PPV), and negative predictive value (NPV).

Of note, 746 time series were constant (e.g., when there were no missing values at all in the data field), and these were included in order that the accuracy reported be representative of the range and distribution of time series across all the data fields.

#### Tuning of consensus algorithm

The *dbscan* package in R accepts 2 tuning parameters: *minPts* (the minimum number of lines needed to create a cluster) and *eps* (the maximum distance between 2 lines in a cluster). In addition, there was the choice of whether or not to include the yellow (uncertain) lines that volunteers had drawn. Therefore, a grid search of 3 parameters was conducted:

- include/exclude yellow (uncertain) lines,
- *minPts* (i.e., minimum-lines-in-cluster) between 2 and 20, and
- *eps* (i.e., epsilon-neighbourhood) between 1px and 7px (i.e., the “minimum distance cutoff”).

Given the imbalanced distribution of positive versus negative calls, Matthews correlation coefficient (MCC) [10] was used to select the highest-performing parameters,


\begin{eqnarray*} MCC\ = \ \frac{{TP \times TN - FP \times FN}}{{\sqrt {\left( {TP + FP} \right) \times \left( {TP + FN} \right) \times \left( {TN + FP} \right) \times \left( {TN + FN} \right)} }} \end{eqnarray*}

where TP = true positives, TN = true negatives, FP = false positives, and FN = false negatives.

### Results

A total of 48,533 classifications were completed by at least 543 different volunteers across the 956 images. After removing repeat classifications by the same person as well as classifications above the retirement threshold of 41, there were 43,502 distinct classifications, and 840 of 956 (88%) images had the full complement of 41 classifications each.

The expert classified each image once, drawing 1,992 green lines plus 163 yellow lines altogether.

**Figure 7:**
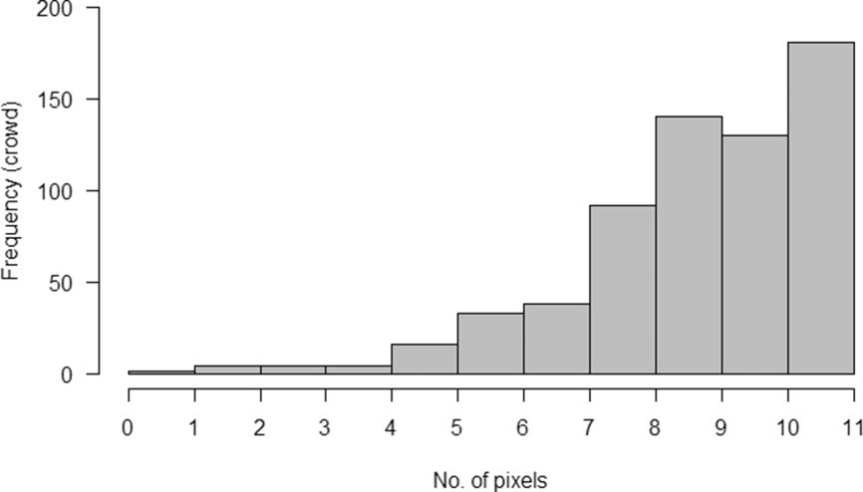
Minimum distances between 2 lines drawn on an image by the same volunteer. Shown up to a maximum of 10px. Intervals are closed on the left and open on the right (i.e., when the minimum distance is an integer, this is included in the bar to the right).

The minimum distance between 2 lines drawn on a single image by the same volunteer was below 1px (see Fig. 7). Since there was a visible threshold in minimum distances at 7px, this was chosen to be the “minimum distance cutoff” for 2 distinct change points. This led to the removal of 96 (0.1%) volunteer lines (with distance <7px) and, for consistency, the removal of 12 (0.6%) expert lines.

Based on the MCC and only using the tuning set of 670 images, the optimal parameters to identify individual change points were as follows: exclude yellow lines, minimum-lines-in-cluster = 5, and epsilon-neighbourhood = 3 (see Table 4), although it should be noted that several different parameter combinations gave very similar performance.

**Table 4:** Best-performing parameters for the density-based clustering algorithm, based on the tuning set

Include yellow lines	Minimum No. of lines in cluster	Epsilon distance	Matthews correlation coefficient	Sensitivity	Specificity	Positive predictive value	Negative predictive value
FALSE	5	3	0.851	0.806	0.999	0.903	0.997
TRUE	7	2	0.850	0.796	0.999	0.913	0.997
TRUE	6	2	0.850	0.826	0.998	0.878	0.997
TRUE	8	3	0.849	0.796	0.999	0.911	0.997
TRUE	7	3	0.848	0.826	0.998	0.875	0.997
FALSE	6	3	0.845	0.769	0.999	0.933	0.996
FALSE	5	2	0.844	0.780	0.999	0.918	0.996
TRUE	6	3	0.844	0.855	0.997	0.838	0.998
FALSE	4	3	0.843	0.843	0.997	0.848	0.997
TRUE	9	3	0.843	0.767	0.999	0.930	0.996

Note: Results are presented as proportions.

#### Final performance of algorithm

Using these parameters on the reserved test set of 286 images resulted in final sensitivity of 80.4% (95% CI, 77.1%–83.3%), specificity of 99.8% (99.7%–99.8%), PPV of 84.5% (81.4%–87.2%), NPV of 99.7% (99.6%–99.7%), and MCC of 0.822. This was from 492 true positives, 38,194 true negatives, 90 false positives (in 42 distinct images), and 120 false negatives (in 70 distinct images).

#### Examples of discrepancies

**Figure 8:**
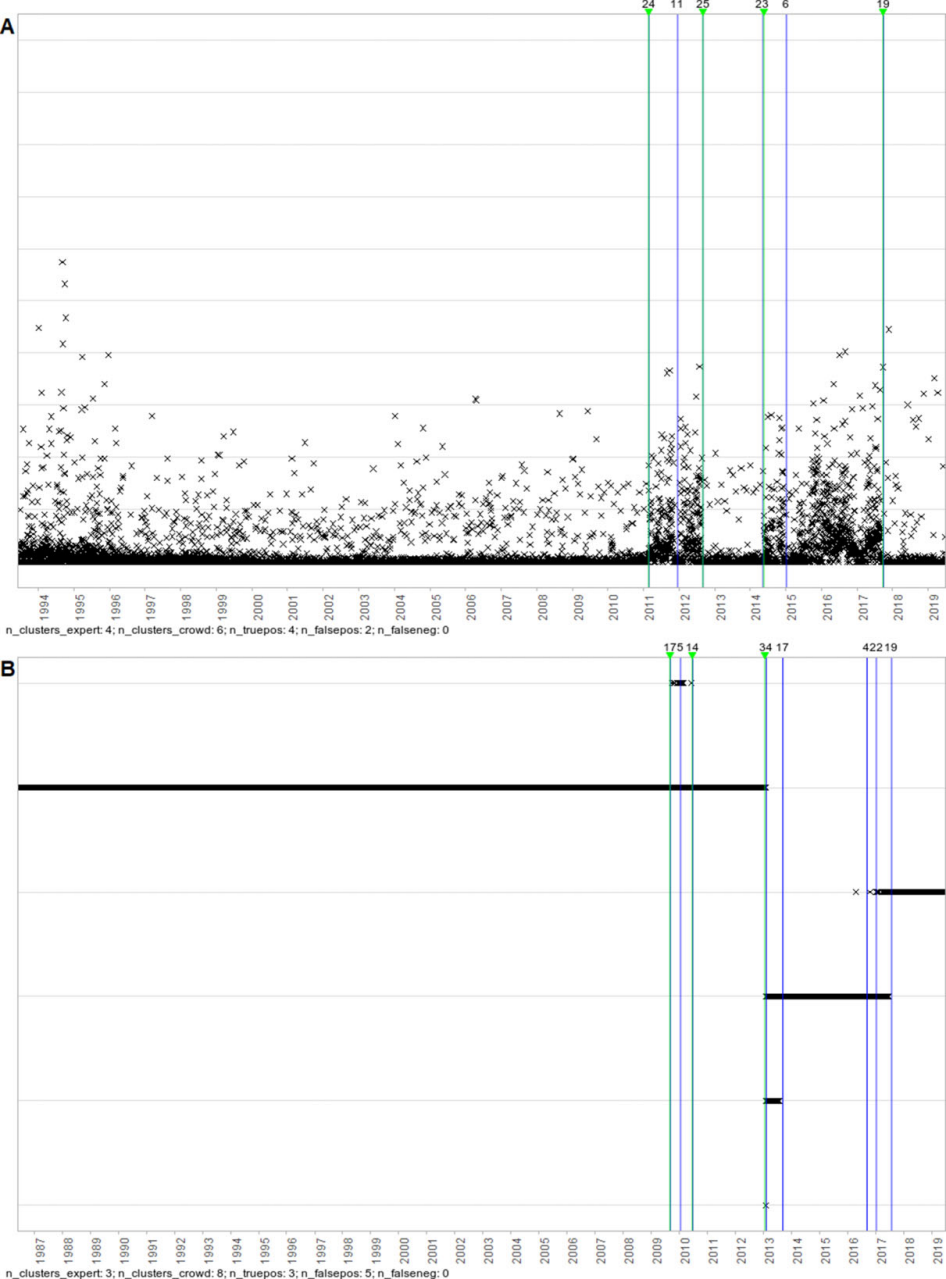
Examples of change points identified by the volunteers but not by the expert. Vertical lines denote positions of volunteer clusters and expert labels; those with numbers above indicate the number of volunteers contributing to the cluster, and those with inverted triangles indicate lines drawn by the expert. (A) The 2 false-positive change points at 2012 and 2015 could arguably be changes in variability. (B) The false-positive change point at 2010 potentially just comprised border points for the 2 adjacent clusters, while the 4 on the far right are likely only related to discretisation.

**Figure 9:**
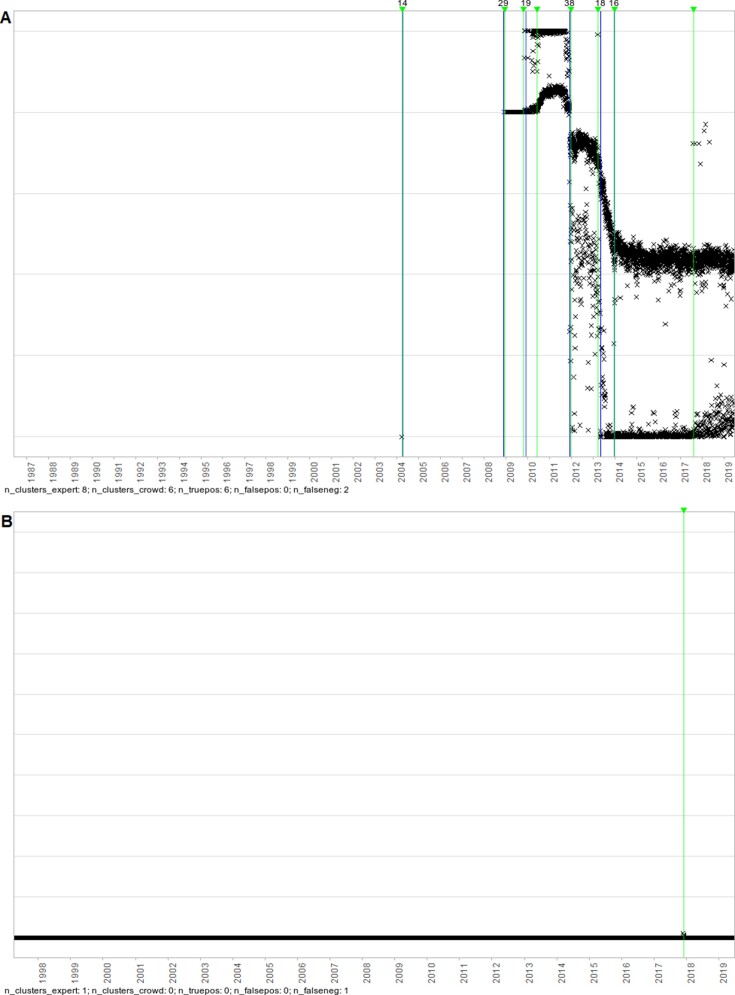
Examples of change points identified by the expert but not by the volunteers. Vertical lines denote positions of volunteer clusters and expert labels; those with numbers above indicate the number of volunteers contributing to the cluster, and those with inverted triangles indicate lines drawn by the expert. (A) The 2 false-negative change points in 2010 and 2017 could arguably be changes in trend or variability. (B) The false-negative change point around 2018 is an outlier that was small in magnitude.

Of the 120 false negatives, 78 (65%) had been classed as clear change points by the expert and 42 (35%) as uncertain. In a random sample of 20 images that contained discrepancies (10 that contained at least 1 false positive and 10 that contained at least 1 clear false negative), there were 30 false positives and 14 false negatives. Twenty-five of 30 of the false positives were in images where the aggregation function values were highly discretised. Seventeen of 30 could be argued to be change points (13 in variability, 3 in trend, 1 outlier), and 1 was in between 2 nearby (true positive) clusters and so potentially merely comprised border points that could have belonged to either of the nearby clusters. Twelve had no explanation beyond the discretisation. Of the 14 false negatives, 7 could be argued to be change points (5 in trend, 1 in variability, 1 outlier), and the other 7 were clear outliers (3 of which were very small in magnitude). See Figures 8 and 9 for examples.

## Discussion and Reuse Potential

Our motivating purpose for releasing this dataset is to improve research quality by encouraging the creation of methods to help screen for temporal artefacts ahead of formal statistical analyses, a highly underappreciated yet important part of the research process [3, 11]. Automating this task will become increasingly valuable as datasets continue to grow (and the effort required to manually check them also increases), whether that be within health research or in other fields that use temporal data.

The primary audience for this dataset, therefore, is developers of (univariate) change point detection methods, who belong to a very active research field [4, 12–14] but for whom there are currently very little real-world data available to either train or validate their methods. In order to assess a detection method's accuracy, a collection of time series containing “ground-truth” labels for the locations of all change points is needed. Synthetic data are commonly used for this task [15, 16], since large numbers of time series with known frequency and locations of change points can be generated easily by concatenating segments from parametric or other statistical models. However, while methods developed and assessed this way may work well for applications where the data happen to conform to the specific models used, they will not work for applications such as ours, where underlying trends and fluctuations in the data are widespread and where the enormous variety of different behaviours exhibited in the different time series is unlikely to be captured by a predefined statistical model.

We are aware of only 3 publicly available time-series datasets that contain real-world data with change points labelled by (expert) humans. These are the Yahoo S5 dataset [17], which contains 67 real-world time series from traffic to Yahoo services; the Numenta Anomaly Benchmark [18], which contains 47 real-world time series from a variety of sources; and the Turing Change Point Dataset [19], which contains 37 time series from a range of different scientific fields. Within all of these, the change points were considered manifestations of real events rather than artefacts of data collection. In comparison, our collection of 5,526 time series provides a vastly larger sample against which to conduct benchmarking of change point detection methods, which will in turn lead to much greater confidence in any results.

Other applications of these change point detection methods could include checking for data feed anomalies in routine analytical pipelines (e.g., the United Kingdom's coronavirus dashboard [20] and Fingertips Public health profiles [21]) in order to alert on any potential data input problems internally before releasing any downstream outputs. Another possible application could be for change detection in automated machine learning (AutoML) models [22, 23], to ensure model validity is maintained even when the data they are being applied to inevitably change.

Crowdsourced labels identifying the locations of change points within EHR time series had a sensitivity of ∼80%, PPV at ∼85%, and specificity/NPV at >99%, when compared to labels made by an experienced data scientist. Given that visual inspection is always going to be a subjective measure, even when performed by an expert, this level of accuracy suggests that crowdsourcing is a satisfactory method for identifying change points in EHR datasets and consequently for use as a “gold standard” to assess automated methods of identifying them.

The types of change points that were most often missed by the volunteers were “outliers” and, to a lesser extent, change points that were small in magnitude. This is potentially acceptable since arguably, outliers are less likely to have a significant impact on a study's results than persistent change points, owing to them occurring for only a small number of records, and similarly change points that are small in magnitude are less likely to have large consequences. Conversely, the volunteers tended to label change points more often than the expert on images based on highly discretised values, which means that certain aggregation functions will likely result in more false-positive calls than others and hence may require more careful scrutiny when being used for tuning automated methods. Many of the discrepancies for the presence of a change point could have been argued either way. This subjectivity means that if these labels are to be used as a “gold standard” for testing automated methods, we can never expect those automated methods to perform perfectly against the labels, and so perhaps we would need to accept a lower accuracy rate than we otherwise would.

The number of change points identified by crowdsourced visual inspection was incredibly high, with change points detected in all 8 data extracts examined, and in almost every year of data that each extract covered. Studies from France [24] and Spain [25] have also found frequent change points in their EHR-related data, despite being more limited in the types of data fields and aggregation functions examined. Given the high risk that *any* data extract obtained from EHRs will contain temporal change points, there is consequently a real risk of flawed or incorrect research results if researchers do not take appropriate steps to identify them and manage their impact. Any ways that can be found to assist them with this task would therefore be highly beneficial.

## Availability of Source Code and Requirements

The dataset described in this article was produced as part of a PhD project, for which the source code has been made available in a Zenodo repository.

Project name: Data quality in health research: the development of methods to improve the assessment of temporal data quality in electronic health records

Project homepage: https://doi.org/10.5281/zenodo.7327780

Operating system(s): Platform independent

Programming language: R v3.6.3

Other requirements: R packages as listed in renv.lock file

License: MIT

## Supplementary Material

giad060_GIGA-D-23-00023_Original_Submission

giad060_GIGA-D-23-00023_Revision_1

giad060_GIGA-D-23-00023_Revision_2

giad060_GIGA-D-23-00023_Revision_3

giad060_Response_to_Reviewer_Comments_Original_Submission

giad060_Response_to_Reviewer_Comments_Revision_1

giad060_Response_to_Reviewer_Comments_Revision_2

giad060_Reviewer_1_Report_Original_Submission Jos Aarts, Ph.D. -- 3/27/2023 Reviewed

giad060_Reviewer_2_Report_Original_Submission Minsu Cho -- 4/19/2023 Reviewed

giad060_Reviewer_2_Report_Revision_1 Minsu Cho -- 5/30/2023 Reviewed

giad060_Reviewer_3_Report_Original_Submission Feng Xie -- 4/19/2023 Reviewed

giad060_Reviewer_3_Report_Revision_1 Feng Xie -- 6/7/2023 Reviewed

## Data Availability

The data set supporting the results of this article is available in the Zenodo repository [26]. All research publications using data derived from Zooniverse [6] approved projects are required to acknowledge the Zooniverse and the Project Builder platform. Please use the text: “This publication uses data generated via the Zooniverse.org platform.”

## Abbreviations

DBSCAN: density-based spatial clustering of applications with noise; DPI: dots per inch; EHR: electronic health record; IORD: Infections in Oxfordshire Research Database; MCC: Matthews correlation coefficient; NHS: National Health Service; NPV: negative predictive value; OUH: Oxford University Hospitals; PNG: Portable Network Graphics; PPV: positive predictive value.
