# Supplementary material for: Health record hiccups—5,526 real-world time series with change points labelled by crowdsourced visual inspection
Source: Gigascience. 2023 Jul 28;12:giad060. doi: 10.1093/gigascience/giad060 (PMC10375518; doi:10.1093/gigascience/giad060)
Supplement: giad060_GIGA-D-23-00023_Original_Submission [file giad060_giga-d-23-00023_original_submission.pdf]

GigaScience

Health Record Hiccups - 5526 real-world time series with change points labelled by crowd-sourced visual inspection

--Manuscript Draft--

**Manuscript Number:**	GIGA-D-23-00023
**Full Title:**	Health Record Hiccups - 5526 real-world time series with change points labelled by crowd-sourced visual inspection
**Article Type:**	Data Note
**Funding Information:**	NIHR Oxford Biomedical Research Centre — Prof Tim Peto<br>National Institute for Health Research Health Protection Research Unit (NIHR200915) — Prof Ann Sarah Walker
**Abstract:**	Background<br><br>Large routinely-collected data such as electronic health records (EHRs) are increasingly used in research, however the statistical methods and processes used to check such data for temporal data quality issues have not moved beyond manual, ad-hoc production and visual inspection of graphs. With the prospect of EHR data being used for disease surveillance via automated pipelines and public-facing dashboards, automation of data quality checks will become increasingly valuable.<br><br>Findings<br><br>We generated 5526 time series from eight different EHR datasets, and engaged >2000 citizen-science volunteers to label the locations of all suspicious-looking change points in the resulting graphs. Consensus labels were produced using density-based clustering with noise, with validation conducted against 956 images containing labels produced by an experienced data scientist. Parameter tuning was done against 670 images, and performance calculated against 286 images, resulting in final sensitivity of 80.4% (95% CI 77.1, 83.3), specificity 99.8% (99.7, 99.8), positive predictive value 84.5% (81.4, 87.2), negative predictive value 99.7% (99.6, 99.7). 12,745 change points were found within 3687 of the time series.<br><br>Conclusions<br><br>This large collection of labelled EHR time series can be used to validate automated methods for change point detection in real-world settings, encouraging the development of methods that can successfully be applied in practice. It is particularly valuable since change point detection methods are typically validated using synthetic data, so their performance in real-world settings cannot be assumed to be comparable. While the dataset focusses on EHRs and data quality, it should also be applicable in other fields.
**Corresponding Author:**	Thai Phuong Quan<br>University of Oxford<br>Oxford, UNITED KINGDOM
**Corresponding Author Secondary Information:**	
**Corresponding Author's Institution:**	University of Oxford
**Corresponding Author's Secondary Institution:**	
**First Author:**	Thai Phuong Quan
**First Author Secondary Information:**	
**Order of Authors:**	Thai Phuong Quan<br>Ben Lacey

	Tim Peto
	Ann Sarah Walker
**Order of Authors Secondary Information:**	
**Additional Information:**	
**Question**	**Response**
Are you submitting this manuscript to a special series or article collection?	No
**Experimental design and statistics**<br><br>Full details of the experimental design and statistical methods used should be given in the Methods section, as detailed in our Minimum Standards Reporting Checklist. Information essential to interpreting the data presented should be made available in the figure legends.<br><br>Have you included all the information requested in your manuscript?	Yes
**Resources**<br><br>A description of all resources used, including antibodies, cell lines, animals and software tools, with enough information to allow them to be uniquely identified, should be included in the Methods section. Authors are strongly encouraged to cite Research Resource Identifiers (RRIDs) for antibodies, model organisms and tools, where possible.<br><br>Have you included the information requested as detailed in our Minimum Standards Reporting Checklist?	Yes
**Availability of data and materials**<br><br>All datasets and code on which the conclusions of the paper rely must be either included in your submission or deposited in publicly available repositories (where available and ethically appropriate), referencing such data using	Yes

Powered by Editorial Manager® and ProduXion Manager® from Aries Systems Corporation

a unique identifier in the references and in the “Availability of Data and Materials” section of your manuscript.<br><br>Have you have met the above requirement as detailed in our Minimum Standards Reporting Checklist?	

Manuscript
Click here to access/download;Manuscript;Data paper.docx

Health Record Hiccups - 5526 real-world time series with change points labelled by crowd-sourced visual inspection

T. Phuong Quan[a], phuong.quan@ndm.ox.ac.uk (corresponding author)

Room 7724, John Radcliffe Hospital, Headley Way, Oxford, OX3 9DU, UK

Ben Lacey[b], ben.lacey@ndph.ox.ac.uk

Timothy E. A. Peto[a], tim.peto@ndm.ox.ac.uk

A. Sarah Walker[a], sarah.walker@ndm.ox.ac.uk

[a]Nuffield Department of Clinical Medicine, University of Oxford, Oxford, UK

[b]Nuffield Department of Population Health, University of Oxford, Oxford, UK.

Abstract

Background: Large routinely-collected data such as electronic health records (EHRs) are increasingly used in research, however the statistical methods and processes used to check such data for temporal data quality issues have not moved beyond manual, ad-hoc production and visual inspection of graphs. With the prospect of EHR data being used for disease surveillance via automated pipelines and public-facing dashboards, automation of data quality checks will become increasingly valuable.

Findings: We generated 5526 time series from eight different EHR datasets, and engaged >2000 citizen-science volunteers to label the locations of all suspicious-looking change points in the resulting graphs. Consensus labels were produced using density-based clustering with noise, with validation conducted against 956 images containing labels produced by an experienced data scientist. Parameter tuning was done against 670 images, and performance calculated against 286 images, resulting in final sensitivity of 80.4% (95% CI 77.1, 83.3), specificity 99.8% (99.7, 99.8), positive predictive value 84.5% (81.4, 87.2), negative predictive value 99.7% (99.6, 99.7). 12,745 change points were found within 3687 of the time series.

Conclusions: This large collection of labelled EHR time series can be used to validate automated methods for change point detection in real-world settings, encouraging the development of methods that can successfully be applied in practice. It is particularly valuable since change point detection methods are typically validated using synthetic data, so their performance in real-world settings cannot be assumed to be comparable. While the dataset focusses on EHRs and data quality, it should also be applicable in other fields.

Keywords

Time series, change point detection, anomalies, data quality

Data description

Context

The use of electronic health records (EHRs) in medical research has grown enormously over the past 20 years, given its ability to cover large numbers of patients and often over long time periods. However, routinely-collected data such as EHRs contain data that was collected for a different purpose (i.e. operational rather than research), and usually at a great distance (both temporally and physically) from the researchers making use of it, so there is an additional need for the data to be checked for unexpected behaviour before any analyses are conducted and conclusions drawn. Events such as changes in data classification (e.g. whether patients undergoing haemodialysis are recorded as inpatients or not), major and minor software updates (e.g. when the available values of a dropdown menu change), or when the meaning or content of a particular data field changes (e.g. when laboratory methods and/or units of measurement are altered), see Figure 1, can lead to sudden changes in the data that, if not identified and taken into account, can lead to erroneous results, incorrect conclusions being drawn, and ultimately result in poor decisions at a clinical or public health policy level.

[Figure 1]

While these temporal artefacts should in theory be picked up by the diligent researcher at the initial data analysis stage, in practice it is not clear to what extent this is actually done, since this process is rarely, if ever, reported in published papers. Standard checks such as the calculation of summary statistics and visual inspection of graphs may be effective enough for traditional research studies where there is a limited number of variables of interest as well as a researcher with appropriate domain knowledge, but with the increasing volume of data being collected in EHRs, and across multiple sites (each with their own idiosyncratic processes), these checks will become more and more onerous and therefore less likely to be conducted thoroughly and consistently. Therefore, automation of checks that would otherwise be labour-intensive and repetitive, such as screening

time series for change points, would be of value to researchers. Furthermore, there is an increasing prospect of EHR data being used for disease surveillance via automated pipelines and public-facing dashboards, where automation of data quality checks will be of even more value.

While there is a rich literature on change point detection methods, with applications across a range of different scientific fields, none of these has to our knowledge been developed with a focus on EHRs or on data quality. Additionally, the vast majority of these methods are validated using synthetic data, and as such their advertised performance cannot be assumed to hold in real-world scenarios. Therefore, in order to assess whether or not any of these methods would be effective to use as a screening method for identifying change points in EHRs requires real-world datasets with "gold standard" labels against which to judge performance.

Methods

Study sample

EHR data is collected from all patients attending the four hospitals within the Oxford University Hospitals NHS Foundation Trust (OUH), which provide all acute care and all microbiology and pathology services in the region (~600,000 individuals). Much of this data is automatically fed into a linked database for use in surveillance and service activities within the OUH, and is periodically extracted into a partially-curated, anonymised, research database, the Infections in Oxfordshire Research Database (IORD). This data goes back to the 1980s and is known to cover multiple periods of change in the hospital computer and laboratory systems.

IORD has Research Ethics Committee and Health Research Authority approval as a generic de-identified electronic research database (19/SC/0403, 19/CAG/0144).

Data was included from all four major component datasets of IORD (patient administration, antibiotic prescribing, haematology/biochemistry laboratories, and microbiology laboratories). Eight

data extracts were taken, comprising a total of 253 data fields and 57 million records, with dates between 2 June 1986 and 30 June 2019:

- inpatient episodes
- outpatient episodes
- emergency department episodes
- antibiotic prescriptions
- biochemistry creatinine tests (a common biomarker for infection)
- haematology neutrophil counts (a standard test requested for most patients)
- microbiology blood culture tests
- microbiology tests that identified *Esherichia coli* (regardless of specimen type)

Creation of time series

A total of 5526 time series were generated from the 8 data extracts, as follows.

One data field from each data extract was selected to be its "**timepoint**" field, and this was used to represent the date of the record (patient administration data used the discharge date, laboratory data used the specimen collection date, and antibiotic data used the prescription date). Any records which contained a missing or invalid datetime value in the timepoint field were necessarily excluded. Also, any duplicate records were removed, and the number of removed records stored as a calculated field.

Aggregation granularities

For each data extract, the time span that each timepoint field covered was divided into regular intervals. Records were aggregated using the chosen timepoint field by **day** (midnight to midnight), as well as by **week** (Monday to Sunday) and by calendar **month**.

Aggregation functions

Numeric summary values were calculated for each timepoint from the (often non-numeric) data by applying simple functions (e.g. number of values present, percentage of missing values, number of distinct values, or median value). If there were no records in a particular timepoint (which meant that no summary value could be calculated), the value of NA was given (except for the aggregation function counting the number of values present in a data field, which would take the value of 0 as expected). Each aggregation function demonstrated a measure within one of the intrinsic data quality dimensions of Completeness, Conformance, and Plausibility[1]. Different functions were used depending on the type of data field:

- **Timepoint –** The data field representing the date of the event described in the record
- **Numeric –** Fields containing continuous values (such as blood cell counts) or discrete integers (such as the episode number within an admission spell)
- **Categorical –** Fields containing a finite list of values, which may be stored either as character strings or coded as integers
- **Datetime –** Fields containing dates, with or without a time element
- **UniqueIdentifier –** Fields containing computer generated record identifiers, and may be based on either a numeric or a character data type
- **Freetext –** Unstructured text

or if applied to the data extract as a whole (e.g. calculating the number of duplicate records). See Table 1 for details of the data fields in each data extract, and Table 2 for the list of aggregation functions applied to each data field.

[Table 1]

[Table 2]

Collection of change point labels by visual inspection

Each time series was plotted on a separate graph (with time on the x-axis and the aggregation function value on the y-axis), see Figure 2 for an example. Frequency-based aggregation functions were plotted on a scale always starting at zero and ending no earlier than 10. Percentages were always plotted on a 0-100 scale, and frequencies of subcategories were plotted on the same scale as frequencies for the data field as a whole. All graphs were saved as png files of the same size, i.e. 1000px wide by 666px tall, at a resolution of 96 dpi.

[Figure 2]

Visually-inspected labels for the locations of change points were collected using the Zooniverse (https://www.zooniverse.org) citizen-science platform. The Zooniverse is a free, popular, and well-established online platform for public involvement in research, and has over 2 million registered volunteers who review and participate in multiple projects from astronomy to wildlife surveys to historical transcriptions.

The "Health Record Hiccups" Zooniverse project showed volunteers one image at a time, and asked them to draw a vertical line on the image wherever they saw an abrupt change in the distribution of values, see Figure 3 for a screenshot. They were initially presented with a tutorial which included multiple examples of different ways in which the data can change – namely changes in **level**, **trend**, (vertical) **variability**, **presence/absence** of data points, or (unpredictable) **outliers**. They were asked to draw a green line if they saw a clear change, a yellow line if they were uncertain, or no lines if they saw no abrupt changes. To reduce risk of bias, no metadata was visible at the point of classification.

[Figure 3]

Images were scheduled for retirement once 41 classifications had been completed on them (i.e. once the image had been inspected by 41 different people).

Data cleaning

Due to the way the Zooniverse platform randomises and supplies images to its volunteers, it was possible for the same person to be served the same image more than once, and for images to have more than the specified number of 41 classifications. Therefore, only the first attempt per person per image was kept, up to a maximum of 41 different people per image.

To improve consistency between classifications made by different volunteers using different screen resolutions, a "minimum distance cut-off" of 7px was selected (see Data validation section) to distinguish between distinct change points (i.e. any lines drawn closer together than this should be assumed to represent the same change point). An example of a 7px distance between two lines is shown in Figure 4. Any lines that were drawn by the same person within this "minimum distance cut-off" interval, were combined into a single line located at the mean position of the contributing lines. If any of the combined lines was green (certain), the resulting line was also considered to be green.

[Figure 4]

Creation of consensus labels

To create consensus labels from the volunteers' classifications, the *dbscan*[2] (density-based spatial clustering of applications with noise[3]) package (v1.1-5) in R (v3.6.3) was used to find zero or more clusters of lines within an image. The mean cluster location was assigned to be the crowd-sourced consensus label for the change point, and any lines that were deemed by the package to be noise, were ignored. Following tuning of the *dbscan* algorithm (see Data validation section), the following three parameters were used to create the final labels for the locations of change points:

- exclude yellow (uncertain) lines,
- minimum-lines-in-cluster (i.e. the minimum no. of lines needed to create a cluster) = 5,
- epsilon-neighbourhood (i.e. the maximum distance between two lines in a cluster) = 3px.

The pixel locations of the consensus labels were then converted back to dates. A total of 12,745 change points were found within 3687 of the time series.

Data validation

Methods

Accuracy of the crowd-sourced consensus labels was assessed against expert labels produced for the initial batch of 956 images (inpatient episodes, antibiotic prescriptions, creatinine tests and blood culture tests, aggregated by day). These expert labels were created by a researcher with >8 years' experience compiling and analysing EHR data, and was done using the same interface as the volunteers, but blinded to any of their results.

To improve consistency between classifications made by different volunteers using different screen resolutions, a "minimum distance cut-off" was selected to distinguish between distinct change points (i.e. any lines drawn closer together than this should be assumed to represent the same change point). This was done by calculating the minimum distance between any two lines drawn on a single image by the same volunteer, and the distribution of minimum-distances visually inspected for a threshold.

To calculate the accuracy of the consensus crowd-sourced labels compared to expert labels, a binary classifier was approximated using the following terms:

- **true positive**: a crowd-sourced label is within the "minimum distance cut-off" of an expert line
- **false positive**: a crowd-sourced label is present, but no expert line lies within the "minimum distance cut-off" of it
- **false negative**: an expert line is present, but no crowd-sourced label lies within the "minimum distance cut-off" of it

- **true negative**: total estimated as the number of the “minimum distance cut-off” intervals in an image (i.e. the maximum number of change points that could possibly be identified on a single image) minus the sum of above 3 categories.

In order to avoid double-counting, the following additional rules were enforced:

- when there were ‘x’ crowd-sourced labels close to one expert line, this counted as one true positive and zero false positives
- when there were two expert lines close to one crowd-sourced label, this counted as two true positives and zero false negatives

Tuning of the algorithm to create consensus labels from the crowd-sourced data was done using a random sample of 70% of the 956 images, balanced across the 4 data extracts, with the remaining 30% reserved for final testing of the performance of the algorithm. Final performance was assessed using sensitivity, specificity, positive predictive value (PPV) and negative predictive value (NPV).

Of note, 746 time series were constant, e.g. when there were no missing values at all in the data field, and these were included in order that the accuracy reported be representative of the range and distribution of time series across all the data fields.

Tuning of consensus algorithm

The *dbscan* package in R accepts two tuning parameters: *minPts* (the minimum no. of lines needed to create a cluster) and *eps* (the maximum distance between two lines in a cluster). In addition, there was the choice of whether or not to include the yellow (uncertain) lines that volunteers had drawn. Therefore, a grid search of three parameters was conducted:

- include/exclude yellow (uncertain) lines,
- *minPts* (i.e. minimum-lines-in-cluster) between 2 and 20,
- *eps* (i.e. epsilon-neighbourhood) between 1px and 7px (i.e. the “minimum distance cut-off”).

Given the imbalanced distribution of positive versus negative calls, Matthew's Correlation Coefficient[4] (MCC) was used to select the highest performing parameters,

$$MCC = \frac{TP \times TN - FP \times FN}{\sqrt{(TP + FP) \times (TP + FN) \times (TN + FP) \times (TN + FN)}}$$

where TP = True Positives, TN = True Negatives, FP = False Positives, FN = False Negatives.

Results

A total of 48,533 classifications were completed by at least 543 different volunteers across the 956 images. After removing repeat classifications by the same person as well as classifications above the retirement threshold of 41, there were 43,502 distinct classifications, and 840/956 (88%) images had the full complement of 41 classifications each.

The expert classified each image once, drawing 1992 green lines plus 163 yellow lines altogether.

The minimum distance between two lines drawn on a single image by the same volunteer was below 1px, see Figure 5. Since there was a visible threshold in minimum-distances at 7px, this was chosen to be the "minimum distance cut-off" for two distinct change points. This led to the removal of 96 (0.1%) volunteer lines (with distance <7px), and for consistency, the removal of 12 (0.6%) expert lines.

[Figure 5]

Based on the MCC and only using the tuning set of 670 images, the optimal parameters to identify individual change points were: exclude yellow lines, minimum-lines-in-cluster = 5 and epsilon-neighbourhood = 3, see Table 3, although it should be noted that several different parameter combinations gave very similar performance.

Table 3. Best performing parameters for the density-based clustering algorithm, based on the tuning set

Include yellow lines	Minimum no. of lines in cluster	Epsilon distance	Matthews Correlation Coefficient	Sensitivity	Specificity	Positive Predictive Value	Negative Predictive Value
FALSE	5	3	0.851	0.806	0.999	0.903	0.997
TRUE	7	2	0.850	0.796	0.999	0.913	0.997
TRUE	6	2	0.850	0.826	0.998	0.878	0.997
TRUE	8	3	0.849	0.796	0.999	0.911	0.997
TRUE	7	3	0.848	0.826	0.998	0.875	0.997
FALSE	6	3	0.845	0.769	0.999	0.933	0.996
FALSE	5	2	0.844	0.780	0.999	0.918	0.996
TRUE	6	3	0.844	0.855	0.997	0.838	0.998
FALSE	4	3	0.843	0.843	0.997	0.848	0.997
TRUE	9	3	0.843	0.767	0.999	0.930	0.996

Note: Results are presented as proportions

Final performance of algorithm

Using these parameters on the reserved test set of 286 images resulted in final **sensitivity** of 80.4% (95% CI 77.1, 83.3), **specificity** 99.8% (99.7, 99.8), **PPV** 84.5% (81.4, 87.2), **NPV** 99.7% (99.6, 99.7), and **MCC** 0.822. This was from 492 true positives, 38,194 true negatives, 90 false positives (in 42 distinct images), and 120 false negatives (in 70 distinct images).

Examples of discrepancies

Of the 120 false negatives, 78 (65%) had been classed as clear change points by the expert, and 42 (35%) as uncertain. In a random sample of 20 images which contained discrepancies (10 which contained at least one false positive and 10 which contained at least one clear false negative), there were 30 false positives and 14 false negatives. 25/30 of the false positives were in images where the

aggregation function values were highly discretised. 17/30 could be argued to be change points (13 in variability, 3 in trend, 1 outlier), and one was in between two nearby (true positive) clusters and so potentially was merely comprised of border points that could have belonged to either of the nearby clusters. 12 had no explanation beyond the discretisation. Of the 14 false negatives, 7 could be argued to be change points (5 in trend, 1 in variability, 1 outlier), and the other 7 were clear outliers (3 of which were very small in magnitude). See Figure 6 and Figure 7 for examples.

[Figure 6]

[Figure 7]

Discussion and re-use potential

Crowd-sourced labels identifying the locations of change points within EHR time series had a sensitivity of ~80%, PPV at ~85%, and specificity/NPV at >99%, when compared to labels made by an experienced data scientist. Given that visual inspection is always going to be a subjective measure, even when performed by an expert, this level of accuracy suggests that crowd-sourcing is a satisfactory method for identifying change points in EHR datasets, and consequently for use as a "gold-standard" to assess automated methods of identifying them.

The types of change points which were most often missed by the volunteers were "outliers", and to a lesser extent, change points which were small in magnitude. This is potentially acceptable since arguably, outliers are less likely to have a significant impact on a study's results than persistent change points, owing to them occurring for only a small number of records, and similarly change points that are small in magnitude are less likely to have large consequences. Conversely, the volunteers tended to label change points more often than the expert on images based on highly discretised values, which means that certain aggregation functions will likely result in more false positive calls than others, and hence may require more careful scrutiny when being used for tuning automated methods. Many of the discrepancies for the presence of a change point could have been

argued either way. This subjectivity means that if these labels are to be used as a “gold standard” for testing automated methods, we can never expect those automated methods to perform perfectly against the labels, and so perhaps we would need to accept a lower accuracy rate than we otherwise would.

Given the high number of change points found across the 8 data extracts investigated, it is highly likely that *any* data extract obtained from EHRs will contain temporal change points in metrics relating to data quality. Therefore, it would be beneficial to find ways to help researchers identify them so that they can take appropriate steps to manage their impact.

We are aware of only three publicly-available time series datasets that contain real-world data with change points labelled by (expert) humans. These are the Yahoo S5 dataset[5] which contains 67 real-world time series from traffic to Yahoo services, the Numenta Anomaly Benchmark[6] which contains 47 real-world time series from a variety of sources, and the Turing Change Point Dataset[7] which contains 37 time series from a range of different scientific fields. Within all of these, the change points were considered to be manifestations of real events rather than artefacts of data collection. In comparison, our collection of 5526 time series provides a vastly larger sample against which to conduct benchmarking of automated change point detection methods, which will in turn lead to much greater confidence in any results.

References

1. Kahn, M.G., et al., *A Harmonized Data Quality Assessment Terminology and Framework for the Secondary Use of Electronic Health Record Data.* EGEMS (Wash DC), 2016. **4**(1): p. 1244.
2. Hahsler, M., M. Piekenbrock, and D. Doran, *{dbscan}: Fast Density-Based Clustering with {R}.* Journal of Statistical Software, 2019. **91**(1): p. 1-30.

3. Ester, M., et al., *A density-based algorithm for discovering clusters in large spatial databases with noise*, in *Proceedings of the Second International Conference on Knowledge Discovery and Data Mining*. 1996, AAAI Press: Portland, Oregon. p. 226–231.
4. Chicco, D. and G. Jurman, *The advantages of the Matthews correlation coefficient (MCC) over F1 score and accuracy in binary classification evaluation.* BMC Genomics, 2020. **21**(1): p. 6.
5. Yahoo Research. *S5 - A Labeled Anomaly Detection Dataset*. 2015 12 July 2022]; Available from: https://webscope.sandbox.yahoo.com/catalog.php?datatype=s&did=70.
6. Ahmad, S., et al., *Unsupervised real-time anomaly detection for streaming data.* Neurocomputing, 2017. **262**: p. 134-147.
7. van den Burg, G.J.J. and C.K.I. Williams, *An Evaluation of Change Point Detection Algorithms.* arXiv preprint, 2020.

Data Availability

The data set supporting the results of this article is available in the Zenodo repository, https://doi.org/10.5281/zenodo.7331161.

Availability of source code and requirements

The data set described in this article was produced as part of a PhD project, for which the source code has been made available in a Zenodo repository.

Project name: Data quality in health research: the development of methods to improve the assessment of temporal data quality in electronic health records

Project home page: https://doi.org/10.5281/zenodo.7327780

Operating system(s): Platform independent

Programming language: R v3.6.3

Other requirements: R packages as listed in renv.lock file

License: MIT

Declarations

List of abbreviations

DBSCAN Density-based spatial clustering of applications with noise

DPI Dots per inch

EHR Electronic Health Record

IORD Infections in Oxfordshire Research Database

MCC Matthew's Correlation Coefficient

NHS National Health Service

NPV Negative Predictive Value

OUH Oxford University Hospitals

PNG Portable Network Graphics

PPV Positive Predictive Value

Ethics approval and consent to participate

This study uses aggregated data from the Infections in Oxfordshire Research Database (IORD). IORD has Research Ethics Committee and Health Research Authority approval as a generic de-identified electronic research database (19/SC/0403, 19/CAG/0144).

Consent for publication

Not applicable

Competing interests

The author(s) declare that they have no competing interests

Funding

This work was funded by the National Institute for Health Research Health Protection Research Unit (NIHR) Health Protection Research Unit in Healthcare Associated Infections and Antimicrobial Resistance (NIHR200915), a partnership between the UK Health Security Agency (UKHSA) and the University of Oxford, and supported by the NIHR Oxford Biomedical Research Centre. The views expressed are those of the author(s) and not necessarily those of the NIHR, UKHSA or the Department of Health and Social Care.

Authors' contributions

TPQ designed and conducted all analyses and data collection, with input from ASW and TEAP. TPQ drafted the article, and all authors revised it critically for important intellectual content.

Acknowledgements

This work uses data generated via the Zooniverse.org platform. We would like to thank the Zooniverse team and all the Zooniverse volunteers who donated their time freely and generously.

This work uses data provided by patients and collected by the NHS as part of their care and support. We thank all the people of Oxfordshire who contribute to the Infections in Oxfordshire Research

Database. Research Database Team: L Butcher, H Boseley, C Crichton, DW Crook, DW Eyre, O Freeman, J Gearing (community), R Harrington, K Jeffery, M Landray, A Pal, TEA Peto, TP Quan, J Robinson (community), J Sellors, B Shine, AS Walker, D Waller. Patient and Public Panel: G Blower, C Mancey, P McLoughlin, B Nichols.

We would like to thank Martin Landray for his contribution as a PhD supervisor on this project.

Figure titles and legends

Figure 1. Examples of temporal changes in data caused by updates to infrastructure at Oxford University Hospitals

(A) Total number of inpatient admissions containing multiple diagnosis codes. The jump in records in 2008 was caused by the inclusion of dialysis day-case patients, which were then excluded again in 2012. (B) Emergency Department attendances by referral source. A change in computer systems in 2011 noticeably affected the data recorded, with the 'Other' category temporarily being overrepresented in 2012, and a new, undefined category of '30' appearing thereafter. (C) Lowest creatinine blood test result each day. The bimodal distribution up to 1997 was due to a mixture of units being used, and the drop in values in 2009 was due to a change in testing method and reference range.

Figure 2. Example of a graph generated for visual inspection of change points

Figure 3. Screenshot of Zooniverse project interface

Figure 4. Example of two lines drawn 7px apart

Any lines drawn closer together than this were considered to represent the same change point

Figure 5. Minimum distances between two lines drawn on an image by the same volunteer

Shown up to a maximum of 10px. Intervals are closed on the left and open on the right, i.e. when the minimum distance is an integer, this is included in the bar to the right.

Figure 6. Examples of change points identified by the volunteers but not by the expert

Vertical lines denote positions of volunteer clusters and expert labels; those with numbers above indicate the number of volunteers contributing to the cluster, those with inverted triangles indicate lines drawn by the expert. (A) The two false positive change points at 2012 and 2015 could arguably be changes in variability. (B) The false positive change point at 2010 is potentially just comprised of border points for the two adjacent clusters, while the 4 on the far right are likely only related to discretisation.

Figure 7. Examples of change points identified by the expert but not by the volunteers

Vertical lines denote positions of volunteer clusters and expert labels; those with numbers above indicate the number of volunteers contributing to the cluster, those with inverted triangles indicate lines drawn by the expert. (A) The two false negative change points in 2010 and 2017 could arguably be changes in trend or variability. (B) The false negative change point around 2018 is an outlier that was small in magnitude.

Table 1. Overview of data fields contained in each data extract

Dataset type	Data extract	Data from	Data to	Total no. of data fields[a]	No. of timepoint fields	No. of numeric fields	No. of categorical fields	No. of datetime fields	No. of uniqueidentifier fields	No. of freetext fields
Antibiotics	Antibiotic prescribing	10/06/2008	30/06/2019	27	1	3	9	7	2	3
Patient administration	ED attendances	01/04/2005	30/06/2019	28	1	1	15	6	2	1
Patient administration	Inpatient episodes	01/04/1997	30/06/2019	41	1	2	23	6	4	3
Patient administration	Outpatient episodes	01/04/1997	30/06/2019	35	1	1	21	4	3	3
Biochemistry	Creatinine tests	02/06/1986	30/06/2019	24	1	1	7	5	6	2
Haematology	Neutrophil counts	01/04/1987	30/06/2019	24	1	1	7	5	6	2

Microbiology	Blood cultures	04/06/1993	30/06/2019	37	1	0	18	6	2	8
Microbiology	E. coli isolations	17/05/1993	30/06/2019	37	1	0	18	6	2	8

[a] Includes two calculated fields, for duplicate records and for all data combined

Table 2. The aggregation functions applied to each data field, to produce the time series

Aggregation function (*shorthand label*)	Individual data field type						Across data extract as a whole	
	timepoint	numeric	categorical	datetime	uniqueidentifier	freetext	All data combined	Duplicate records
**COMPLETENESS**								
Number of missing values (*missing_n*)		x	x	x	x	x	x	
Percentage of missing values (*missing_perc*)		x	x	x	x	x	x	
**CONFORMANCE**								
Number of non-conformant values[a] (*nonconformant_n*)		x		x			x	
Percentage of non-conformant values[a] (*nonconformant_perc*)		x		x			x	
**PLAUSIBILITY**								
Sum of duplicate records removed (*sum*)								x
Percentage of records which had been duplicated (*nonzero_perc*)								x

Number of values present (*n*)	x	x	x	x	x	x	x	
Minimum value (*min*)		x		x				
Maximum value (*max*)		x		x				
Mean value (*mean*)		x						
Median value (*median*)		x						
Number of values with no time element[b] (*midnight_n*)	x			x				
Percentage of values with no time element[b] (*midnight_perc*)	x			x				
Minimum string length (*minlength*)					x			
Maximum string length (*maxlength*)					x			
Mean string length (*meanlength*)					x			
Number of distinct values (*distinct*)			x					
Number of values within each subcategory[c] (*subcat_n*)			x					
Percentage of values within each subcategory[c] (*subcat_perc*)			x					

[a] Non-conformance was deemed as a non-numeric value in a (supposedly) numeric data field, or a non-date value in a (supposedly) date field

[b] These were only calculated for fields that were known to contain a time element, and where midnight would be used as the default when no time element was available

[c] With one time series created per subcategory. These were only calculated for fields with fewer than 20 subcategories (with the additional inclusion of DischargeDestinationCode in the inpat_episode data extract, which contained 23 subcategories, and was included for consistency with the other coded fields in the data extract)

Figure 1

Click here to access/download;Figure;Figure 1.png

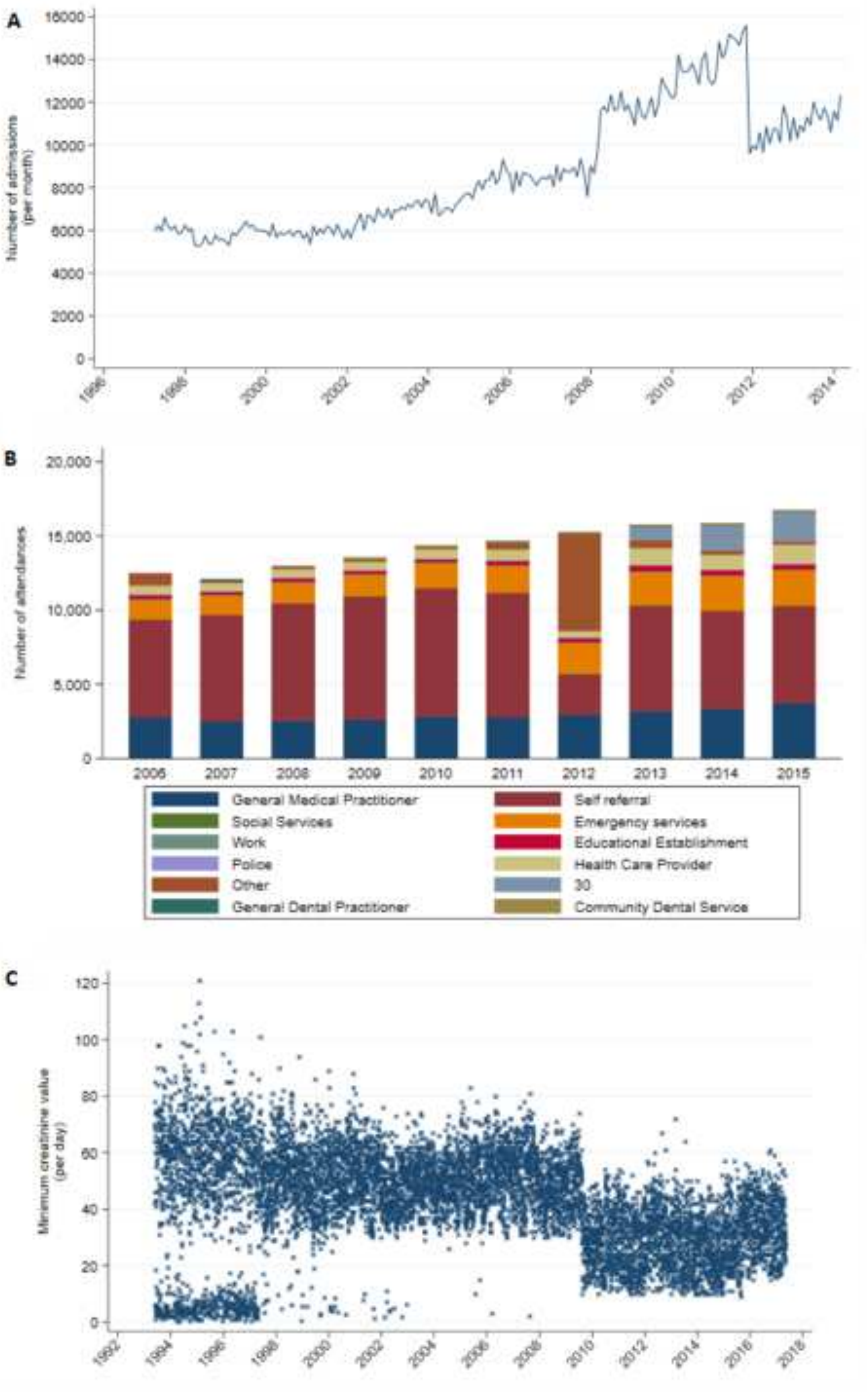

Figure 2

Click here to access/download;Figure;Figure 2.png

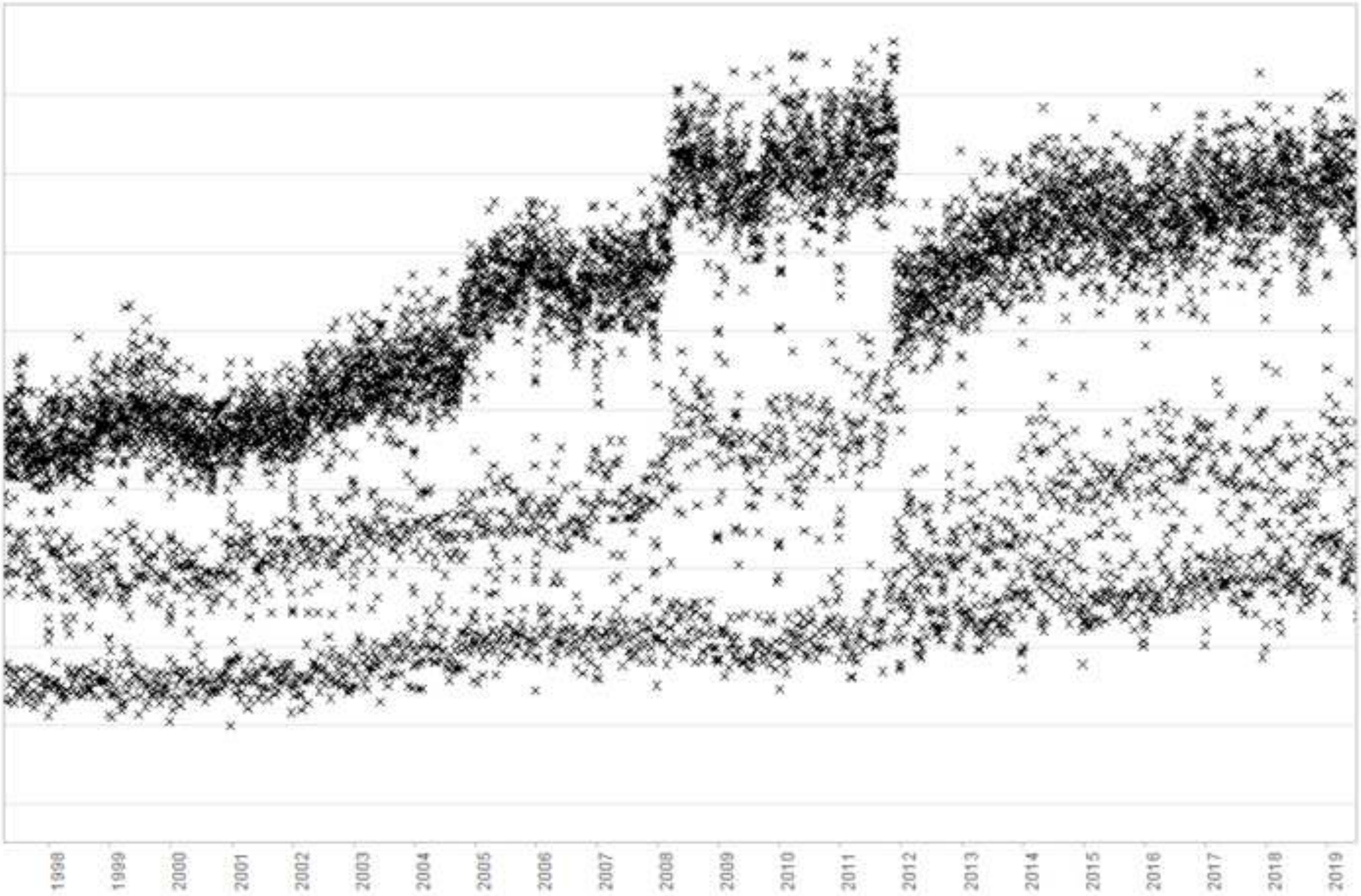

Figure 3

Click here to access/download;Figure;Figure 3.png

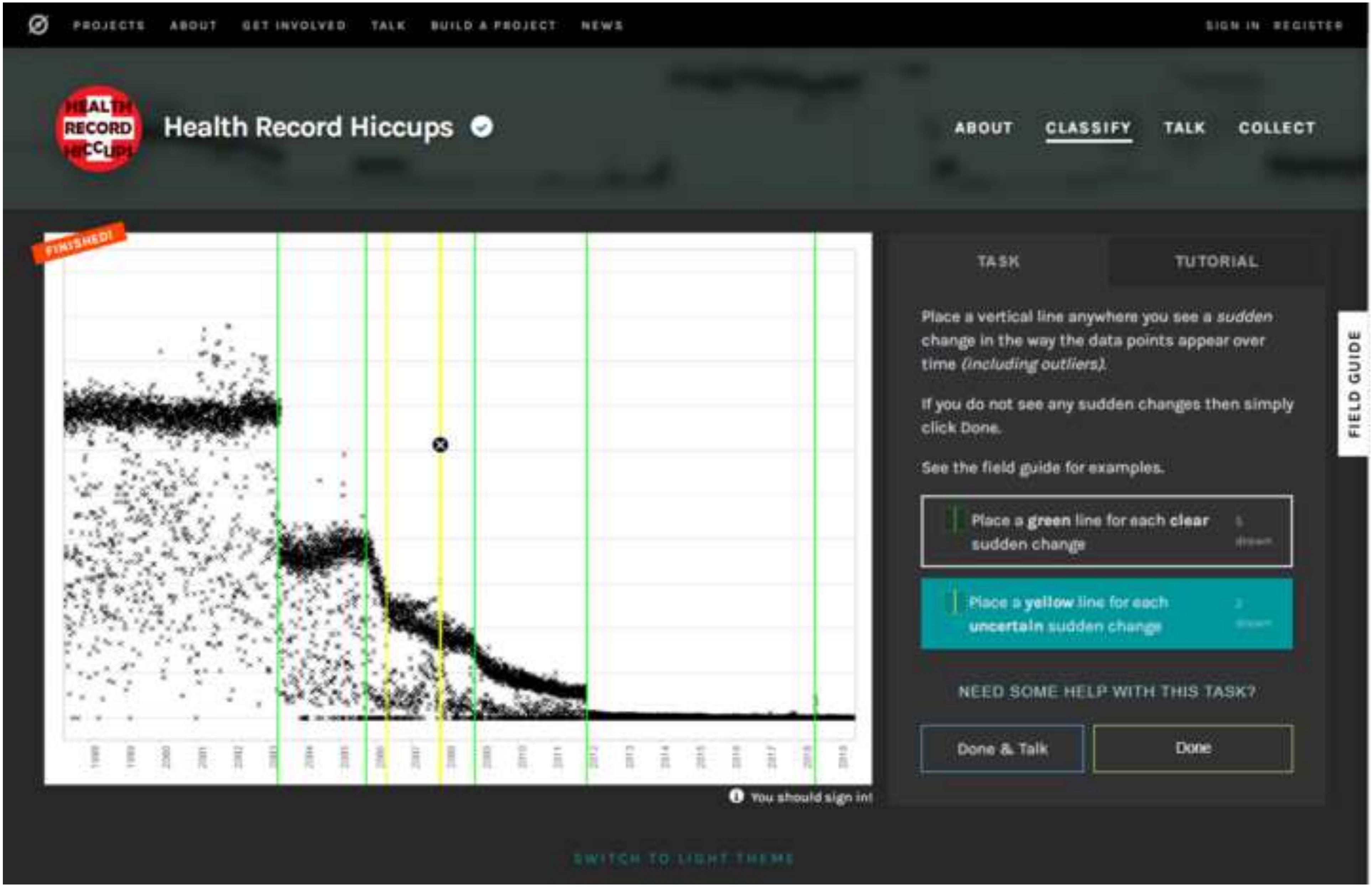

Figure 4

Click here to access/download;Figure;Figure 4.png

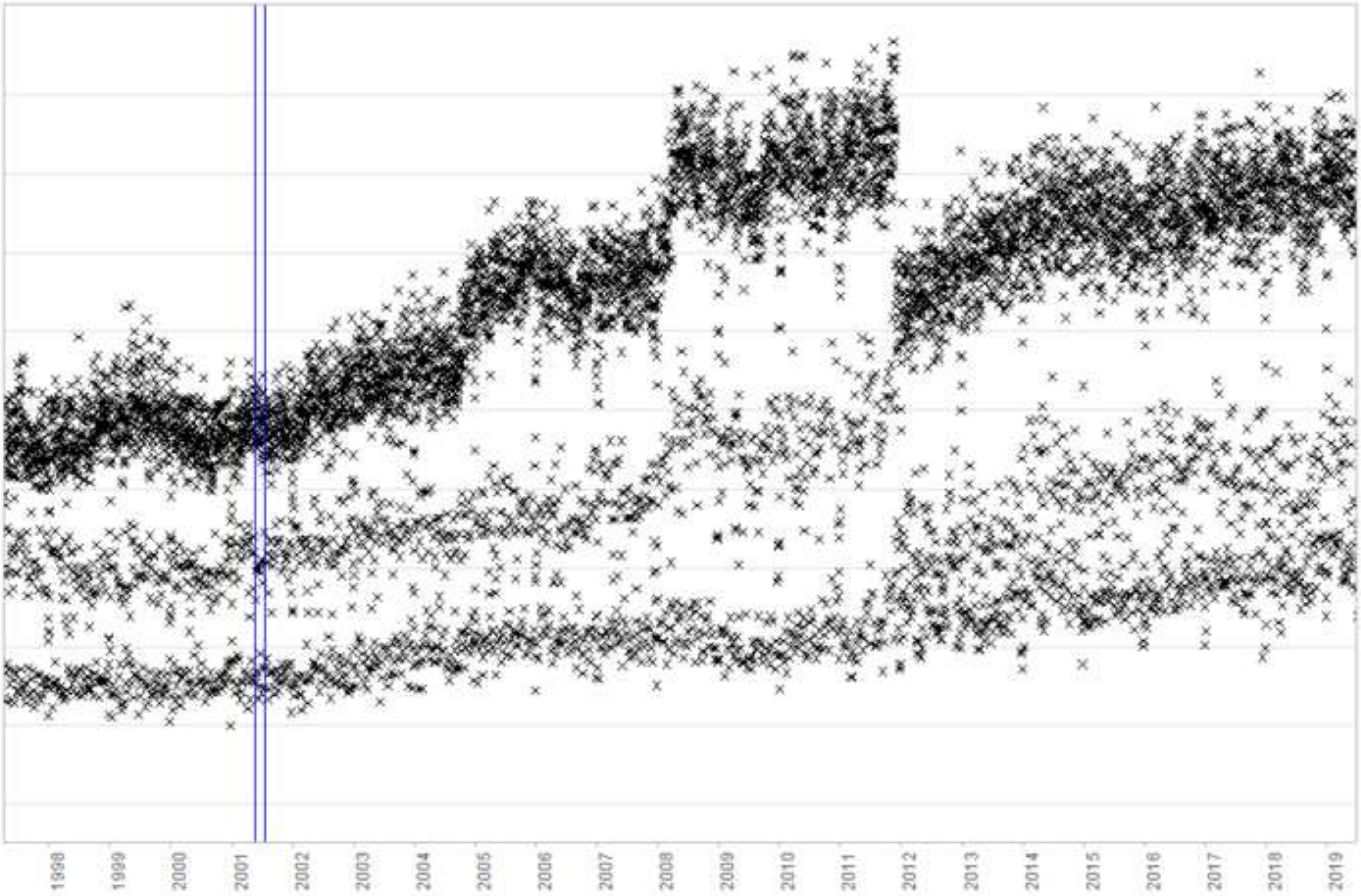

Figure 5

Click here to access/download;Figure;Figure 5.png

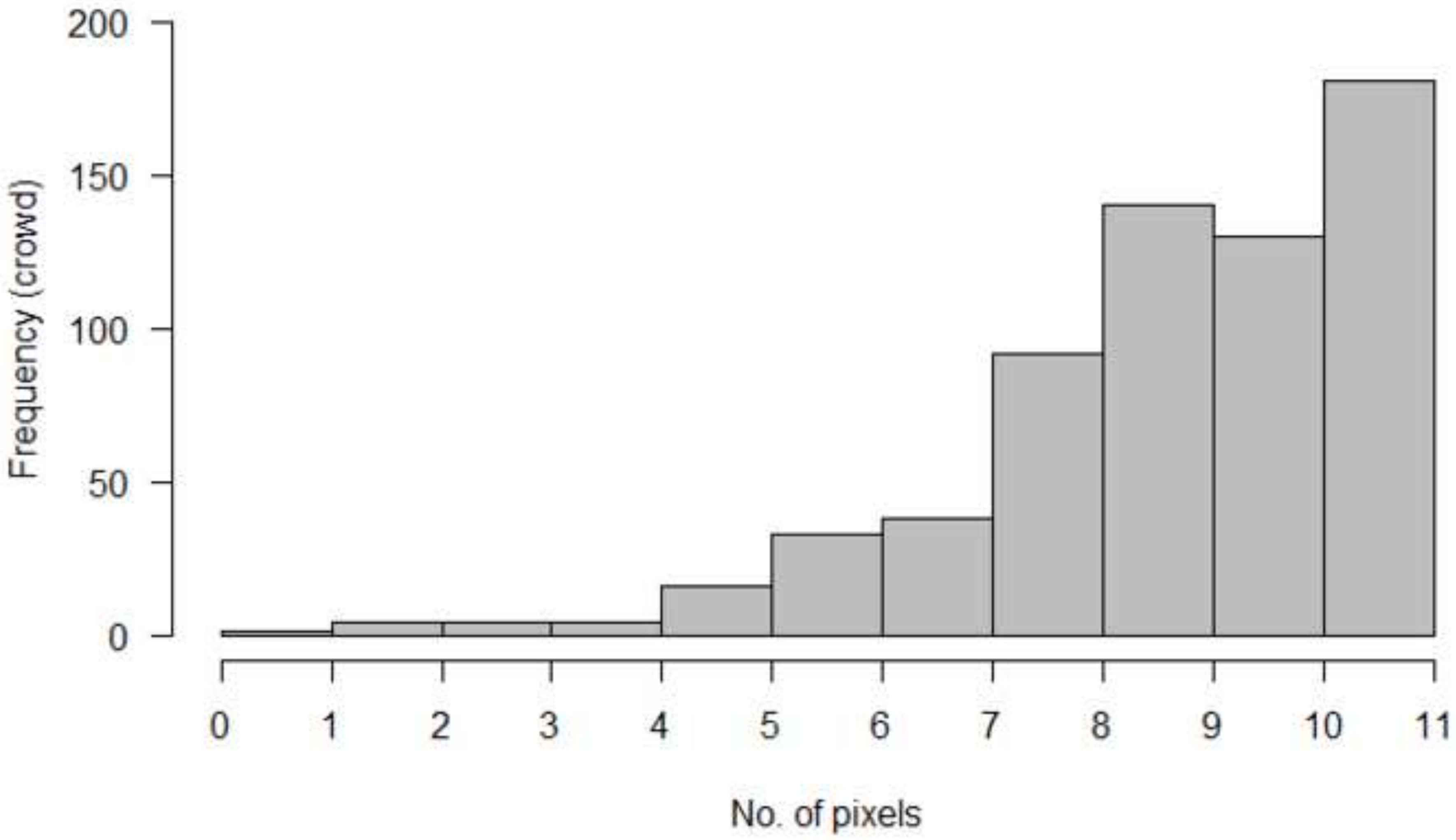

Figure 6

Click here to access/download;Figure;Figure 6.png

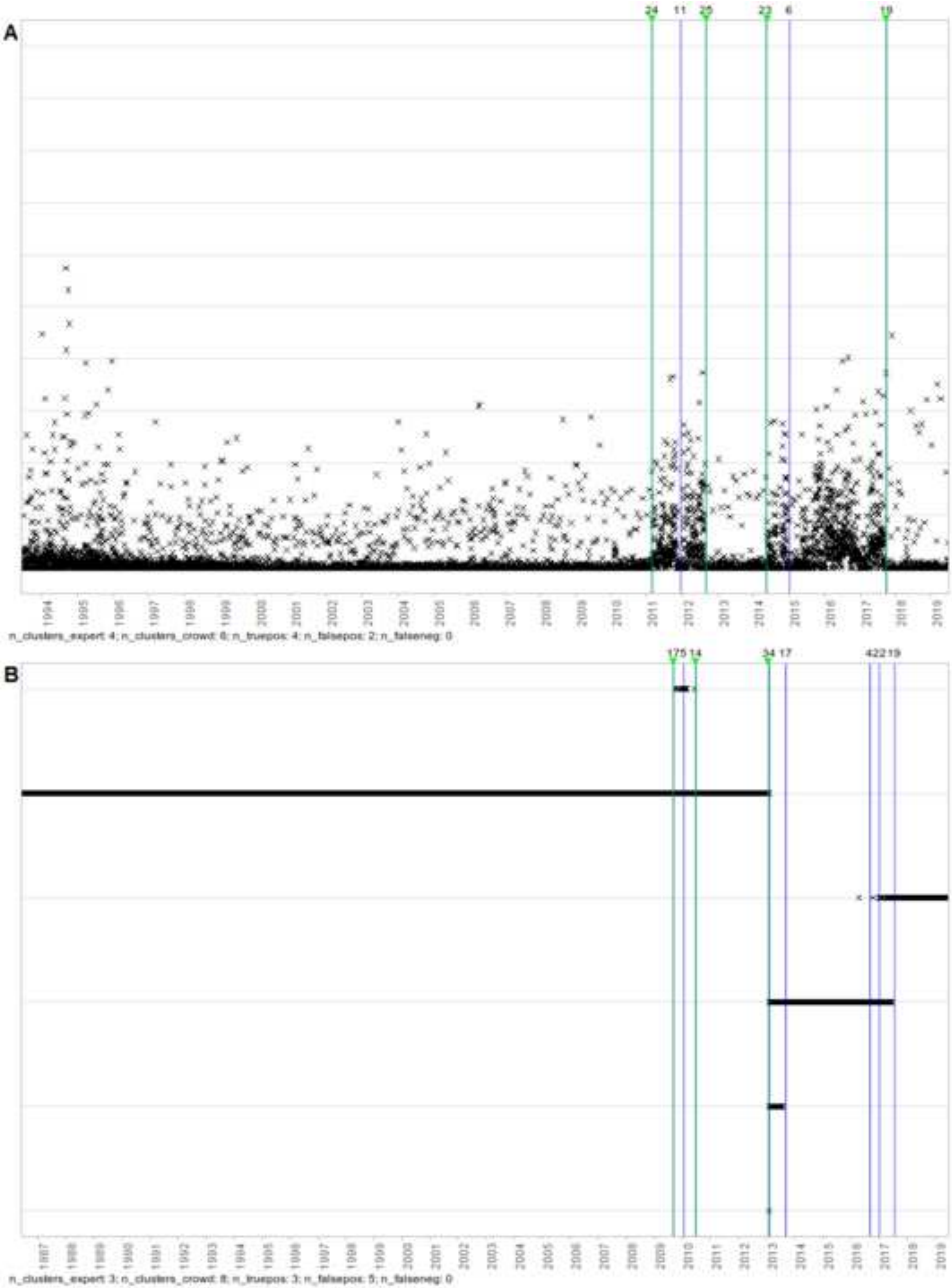

Figure 7

Click here to access/download;Figure;Figure 7.png

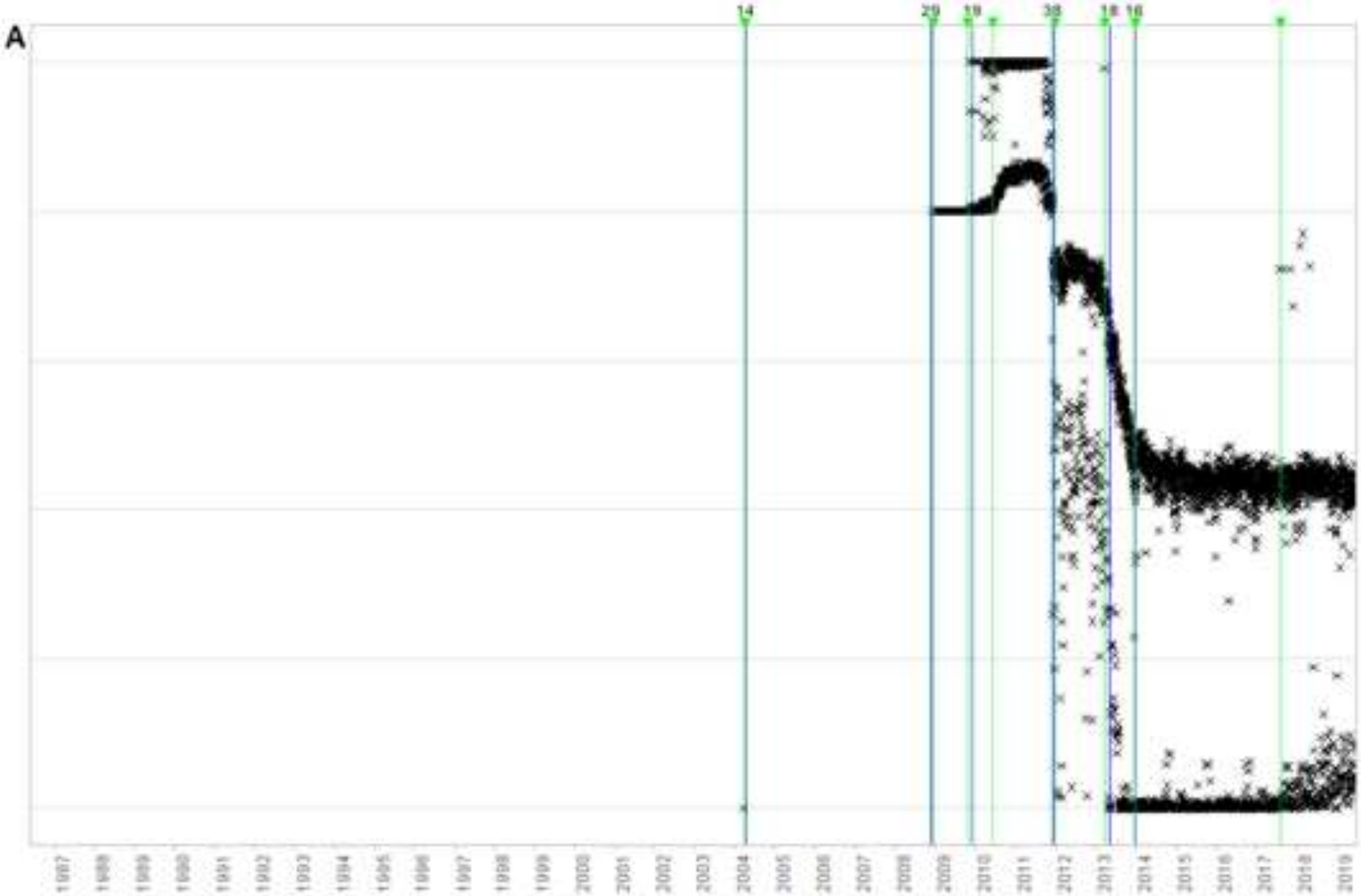

n_clusters_expert: 8; n_clusters_crowd: 0; n_truepos: 0; n_falsepos: 0; n_falseneg: 2

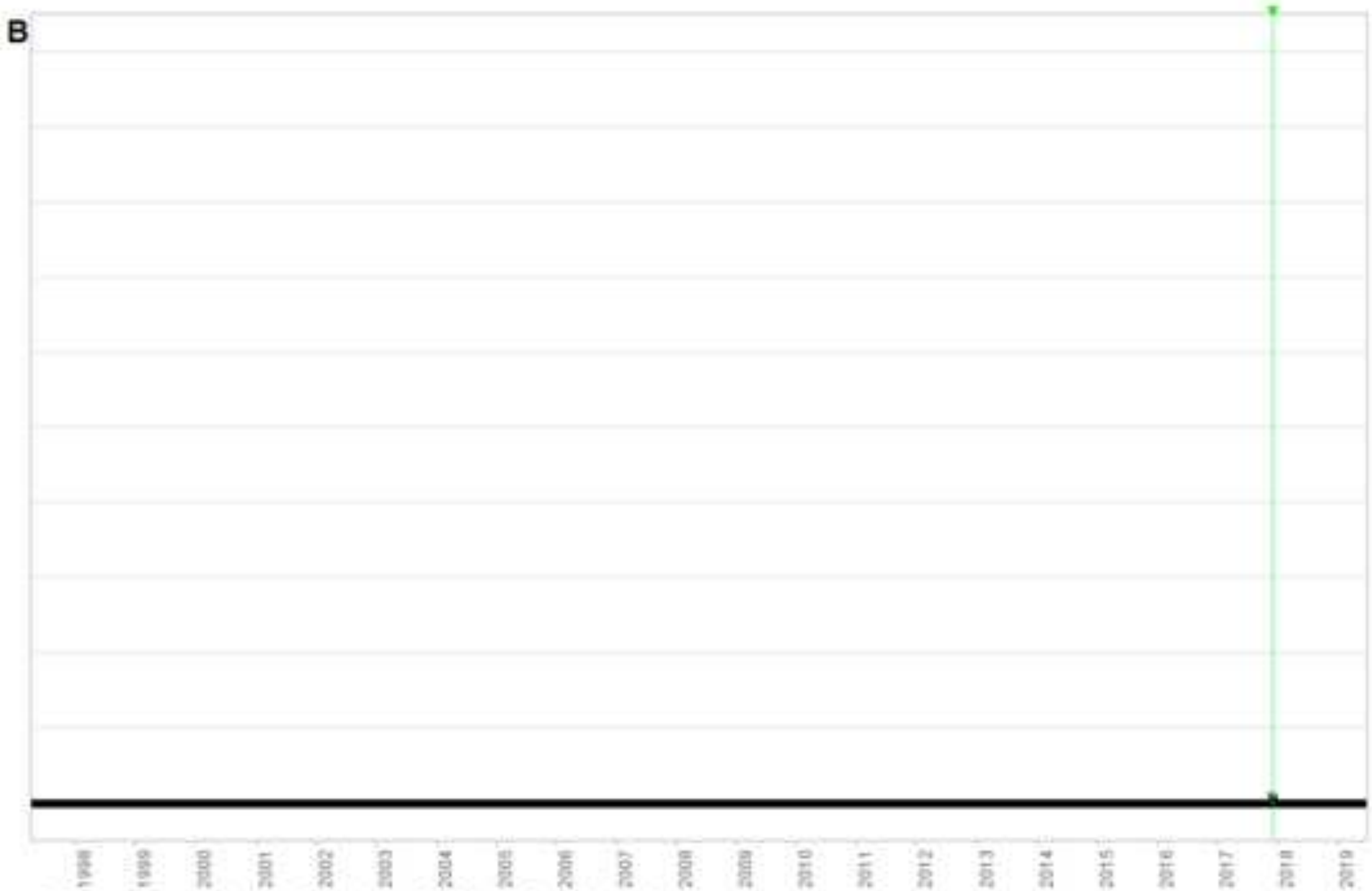

n_clusters_expert: 1; n_clusters_crowd: 0; n_truepos: 0; n_falsepos: 0; n_falseneg: 1
