# Supplementary material for: Health record hiccups—5,526 real-world time series with change points labelled by crowdsourced visual inspection
Source: Gigascience. 2023 Jul 28;12:giad060. doi: 10.1093/gigascience/giad060 (PMC10375518; doi:10.1093/gigascience/giad060)
Supplement: giad060_Response_to_Reviewer_Comments_Original_Submission [file giad060_response_to_reviewer_comments_original_submission.pdf]

Author's Response To Reviewer Comments

Close

GENERAL RESPONSE: We would like to thank the reviewers for their time and feedback. We have responded to the reviewers' questions and made revisions as described below.

Reviewer #1: It is for me unusual to review a paper that is published outside medicine and/or biomedical informatics. The paper stands out for two reasons. First, it looks at a temporal sequence of data extracted from EHRs and second it aims to identify change points that are relevant to include when data are interpreted. Novel is the approach to include citizen-science volunteers. The results are promising. Of course, the hope is that existing algorithms will be improved and better one developed to identify change points. I think including citizen-scientists volunteers in this type of research is intrinsically valuable. The Patient-Centered Outcome Research Institute (PCORI) in Washington DC funds extensively the development of federated databases that span many health care organizations to study patient problems. It aims to involve actively patients in such studies. This approach could be very helpful to involve patients. I suggest therefore that a follow-up paper will be published, aimed at medical researchers, epidemiologists and biomedical informatics researchers, not only to explain the approach, but also its potential and limitations, including the Zooniverse system. I have a few questions.

What are the limitations of the study and the recruitment of citizen-science volunteers?
RESPONSE: One limitation of the dataset is that it comes from a single hospital group, which may lead to questions about generalisability. However, the group includes four distinct hospitals containing multiple different specialties, and a range of hospital activities were covered by the 8 different data extracts. All organisations undergo changes in systems and processes over time, so there is no reason to believe that these types of changes would not also manifest in other EHRs as well.
While the most obvious limitation of using volunteers was that we couldn't ensure that each volunteer had correctly understood the task, this was mitigated by the large number of volunteers whose labels could be pooled, and any outliers excluded. We also describe in the Validation section, situations where the volunteers performed less well. Overall, we found that the Zooniverse platform made it very easy to create and test new projects, and the recruitment of citizen-science volunteers was effortless, but that more time and effort was needed to field volunteers' questions and comments than we had anticipated.

Have problems to be kept simple in order to make participation feasible?
RESPONSE: Yes, the pilot version of the citizen-science project used a more complicated task, where the volunteers were asked to label the "type" of change point as well as its location. However, the results were too inconsistent between volunteers to be usable. Therefore, for the final project (which was used to generate this dataset), the task was simplified to only request the location to be labelled.

Reference 7 is an arXiv preprint allowing for on-line peer review. Has a final version been published in a journal? Actually, arXiv is hotly debated in biomedicine whether it is a valid approach to publish papers before they are accepted in biomedical journals.
RESPONSE: There does not appear to have been a final version published in a journal, as the preprint is still specified as the appropriate citation by the authors of the dataset.

Reviewer #2: This article introduces a large time series dataset from eight different electronic health records that aim to be utilized for detecting a situation where the statistical properties of the clinical data change over time, i.e., concept drift in machine learning. Regarding this, they collected crowd-sourced labels and compared them with the result of experts. Also, they validated their data by employing some evaluation metrics, such as sensitivity, PPV, specificity, and MCC.
This article effectively introduces their dataset and follows the required guideline as a data note article. Also, this reviewer believes that this dataset can contribute to building a time-series model in a clinical setting, deriving how to monitor key performance indicators when operating a healthcare service, and even

identifying general concept drift points.
However, before accepting and publishing this paper, this paper should resolve the following issues.

i) As mentioned, there are different state-of-the-art concept drift techniques in the data science discipline. The authors consider comparing the human-labeled results with the machine-labeled results using the concept drift techniques.
RESPONSE: We are not certain what the reviewer is asking for here and have assumed that the second sentence was supposed to read "The authors [should] consider comparing the human-labeled results with the machine-labeled results...". Comparing machine-labelled results to the human-labelled results is indeed the purpose of making this dataset available, however, we consider such an analysis to be distinct from the creation of the dataset and hence out of scope for inclusion in this paper, whose purpose is to describe the dataset itself.
However, this was explored in Chapter 5 of the wider PhD thesis (https://doi.org/10.5281/zenodo.7327780) from which this dataset forms a part. This analysis compared a range of change point detection techniques to the human-labelled results, all of which performed poorly, with maximum sensitivity of 36.9% (95% CI 35.2, 38.8), specificity of 100% (100, 100), PPV of 51.6% (49.4, 53.8), and NPV of 99.9% (99.9, 99.9).

ii) The dataset only includes the location of the data change as labels. However, we can map data change points into several categories, such as sudden change, blip change, recurring change, etc. Thus, authors need to consider those types of changes to extend the reusability of the data.
RESPONSE: We agree that classifying the "type" of each change point would also have been very useful, and in the pilot version of the citizen-science project, the volunteers were asked to label the change point "type" as well as its location. However, the results were too inconsistent between volunteers to be usable. Therefore, for the final project (which was used to generate this dataset), the task was simplified to only request the location to be labelled.

iii) The authors need to suggest a couple of practical examples for utilizing the dataset in a healthcare setting.
RESPONSE: Our intended purpose for this dataset is to assist the development and validation of change point detection methods, and we anticipate that it is these methods that will be applied in a healthcare setting rather than the dataset itself. We are very happy if readers can envisage other uses for the dataset within their particular domain, but we do not wish to suggest uses outside of our expertise. In terms of using any subsequent change point detection methods, we have added some general examples of uses for these methods in the Discussion, and have also described two motivating examples from a healthcare setting in the Context section.

iv) All figures have poor quality. They need to be improved before publishing.
RESPONSE: We will ensure the figures are of the correct quality for the final version

Reviewer #3: The study by Quan et al. describes a large collection of labeled EHR time series that can be used to validate automated methods for change point detection. There are 5526 time series generated from 8 different EHR datasets, engaging over 2000 citizen-science volunteers. This study tried to present a large collection of labeled EHR time-series for open and reproducible research. However, there are some concerns in terms of the research gap and implications. Some important information might be missing. I have summarized some major points below.

1. I didn't well understand the research gap and unmet need the authors wanted to address. Please address why the change point detection in EHR is important and should be addressed. It would be better to give some real-world examples to let readers have s clear understanding of the importance of your question. The research gap should also be well-identified in the introduction by citing some references. I noticed that few references were cited in the introduction. The authors should add more references to prove that what you present is credible and related to previous research.
RESPONSE: We have largely rewritten paragraph 1, expanding it to highlight the three real-world examples of change points shown in Figure 1, and to make it clearer what the consequences might be if those change points were not identified when using that data. We have also added a paragraph to the end of the Discussion, linking to other studies that have identified change points in EHR-related data (though not by

crowd-sourcing). We have added 4 new references to the introduction and 4 to the Discussion.

2. It would be nice to give a figure to show the whole workflow. I feel there are so many technical details in the manuscript, and the author might want to organize these in clear logic to make your workflow easy to understand.
RESPONSE: We have added a new Figure 2 showing an overview of the workflow.

3. It would be good to present a more overall descriptive analysis of the data to show us the essential characteristics of the information, especially since this is a data descriptor. We care more about what your data look like instead of some technical details on how you extract data.
RESPONSE: We agree that a fuller description of the characteristics of the data will encourage its reuse and so have added a new Table 3 describing some properties of the time series and numbers of change points found within each of the 8 data extracts. We have also split Figure 3 (previously Figure 2) into multiple panels to show more examples of what the individual time series look like, and added a new Figure 6 to show examples of the locations of crowd-sourced labels.

4. I notice some grammar errors, and please proofread to minimize them. Also, the last sentence of the first paragraph is too long.
RESPONSE: We have proofread the manuscript again, and made some edits to improve clarity and shorten sentences.

5. I would suggest adding a substantial paragraph on the implication of your study. It can include how your data can be used in the real world or where future researchers can utilize your data. These should be elaborated on and fully discussed to show that your study/data is of impact.
RESPONSE: We have reorganised and expanded the Discussion to make the purpose and utility of the dataset clearer, adding a paragraph about its importance for improving the real-world validity of change point detection methods, and suggesting further potential applications for those methods.

Close
