# Supplementary material for: Health record hiccups—5,526 real-world time series with change points labelled by crowdsourced visual inspection
Source: Gigascience. 2023 Jul 28;12:giad060. doi: 10.1093/gigascience/giad060 (PMC10375518; doi:10.1093/gigascience/giad060)
Supplement: giad060_Response_to_Reviewer_Comments_Revision_1 [file giad060_response_to_reviewer_comments_revision_1.pdf]

Author's Response To Reviewer Comments

Close

Reviewer #3: Thank the authors for the revision. The response has addressed my concerns. The overall quality and structure have been improved. The logic is clearer now.

One outstanding issue is that I am still not very satisfied with the discussion part. The authors have added more applications to this data and how future researchers can use your data (I feel it's very important as this is a data note, and the main aim is to involve future researchers in using your data). But these are not well elaborated, and the citation/references were not fully provided. For example, when the authors talked about "the primary audience for this dataset is developers of (univariate) change point detection methods", it's better to have some references and citations (what are they?), and talk more about HOW the data can be used, rather than the lack of publicly-available data. Also, when the author mentioned the application of this dataset to AutoML and outlier detection, no references were cited. It's better to cite some papers to show the audience HOW they used the dataset to develop a method or more

Response:
We have reorganised and expanded the first three paragraphs of the discussion and added nine new references.

We have thought hard about the best way to expand on "HOW the data can be used", and we envisage only a very narrow application for the *data* itself, namely as a training and/or test set for the development/validation of change point detection methods (clarified in the revised text). Exactly how to use the dataset to develop/validate a detection method will depend on the specific application, so we do not feel we can provide specific details on this. Instead, we have added new text to elaborate on the necessity of validating change point detection methods against datasets which are representative of the data on which the methods will eventually be applied (rather than synthetic simulated data as is commonly done), and added 5 new references relating to the literature around change point detection methods. Further, we are not able to cite references on "HOW they used the dataset to develop a method or more", as the dataset has not to date been used to develop any new methods. We have however added two new references describing the relevance of change detection to AutoML, and also provided two examples of routine analytical pipelines that could potentially benefit from automated checks for data feed anomalies.

We hope that these additions and clarifications address the reviewer's concerns, but would of course be happy to make further changes if the reviewer or Editors can clarify the specific aspects they feel we are missing.

Lastly, we discovered that there was a more appropriate reference for the Numenta Anomaly Benchmark than the one that was previously used, so have updated it.

Close
