# Supplementary material for: Health record hiccups—5,526 real-world time series with change points labelled by crowdsourced visual inspection
Source: Gigascience. 2023 Jul 28;12:giad060. doi: 10.1093/gigascience/giad060 (PMC10375518; doi:10.1093/gigascience/giad060)
Supplement: giad060_Response_to_Reviewer_Comments_Revision_2 [file giad060_response_to_reviewer_comments_revision_2.pdf]

Author's Response To Reviewer Comments

Close

I have made the requested changes. Many thanks.

Close
