# Supplementary material for: Health record hiccups—5,526 real-world time series with change points labelled by crowdsourced visual inspection
Source: Gigascience. 2023 Jul 28;12:giad060. doi: 10.1093/gigascience/giad060 (PMC10375518; doi:10.1093/gigascience/giad060)
Supplement: giad060_Reviewer_1_Report_Original_Submission — Jos Aarts, Ph.D. -- 3/27/2023 Reviewed [file giad060_reviewer_1_report_original_submission.pdf]

Reviewer Report

Title: Health Record Hiccups - 5526 real-world time series with change points labelled by crowd-sourced visual inspection

Version: Original Submission **Date:** 3/27/2023

Reviewer name: Jos Aarts, Ph.D.

Reviewer Comments to Author:

It is for me unusual to review a paper that is published outside medicine and/or biomedical informatics. The paper stands out for two reasons. First, it looks at a temporal sequence of data extracted from EHRs and second it aims to identify change points that are relevant to include when data are interpreted. Novel is the approach to include citizen-science volunteers. The results are promising. Of course, the hope is that existing algorithms will be improved and better one developed to identify change points. I think including citizen-scientists volunteers in this type of research is intrinsically valuable. The Patient-Centered Outcome Research Institute (PCORI) in Washington DC funds extensively the development of federated databases that span many health care organizations to study patient problems. It aims to involve actively patients in such studies. This approach could be very helpful to involve patients. I suggest therefore that a follow-up paper will be published, aimed at medical researchers, epidemiologists and biomedical informatics researchers, not only to explain the approach, but also its potential and limitations, including the Zooniverse system. I have a few questions. What are the limitations of the study and the recruitment of citizen-science volunteers? Have problems to be kept simple in order to make participation feasible? Reference 7 is an arXiv preprint allowing for on-line peer review. Has a final version been published in a journal? Actually, arXiv is hotly debated in biomedicine whether it is a valid approach to publish papers before they are accepted in biomedical journals.

Level of Interest

Please indicate how interesting you found the manuscript: Choose an item.

Quality of Written English

Please indicate the quality of language in the manuscript: Choose an item.

Declaration of Competing Interests

Please complete a declaration of competing interests, considering the following questions:

- Have you in the past five years received reimbursements, fees, funding, or salary from an organisation that may in any way gain or lose financially from the publication of this manuscript, either now or in the future?

- Do you hold any stocks or shares in an organisation that may in any way gain or lose financially from the publication of this manuscript, either now or in the future?
- Do you hold or are you currently applying for any patents relating to the content of the manuscript?
- Have you received reimbursements, fees, funding, or salary from an organization that holds or has applied for patents relating to the content of the manuscript?
- Do you have any other financial competing interests?
- Do you have any non-financial competing interests in relation to this paper?

If you can answer no to all of the above, write 'I declare that I have no competing interests' below. If your reply is yes to any, please give details below.

I declare that I have no competing interests.

I agree to the open peer review policy of the journal. I understand that my name will be included on my report to the authors and, if the manuscript is accepted for publication, my named report including any attachments I upload will be posted on the website along with the authors' responses. I agree for my report to be made available under an Open Access Creative Commons CC-BY license (http://creativecommons.org/licenses/by/4.0/). I understand that any comments which I do not wish to be included in my named report can be included as confidential comments to the editors, which will not be published.

Choose an item.

To further support our reviewers, we have joined with Publons, where you can gain additional credit to further highlight your hard work (see: https://publons.com/journal/530/gigascience). On publication of this paper, your review will be automatically added to Publons, you can then choose whether or not to claim your Publons credit. I understand this statement.

Yes Choose an item.
