# Supplementary material for: Health record hiccups—5,526 real-world time series with change points labelled by crowdsourced visual inspection
Source: Gigascience. 2023 Jul 28;12:giad060. doi: 10.1093/gigascience/giad060 (PMC10375518; doi:10.1093/gigascience/giad060)
Supplement: giad060_Reviewer_2_Report_Original_Submission — Minsu Cho -- 4/19/2023 Reviewed [file giad060_reviewer_2_report_original_submission.pdf]

Reviewer Report

Title: Health Record Hiccups - 5526 real-world time series with change points labelled by crowd-sourced visual inspection

Version: Original Submission **Date:** 4/19/2023

Reviewer name: Minsu Cho

Reviewer Comments to Author:

This article introduces a large time series dataset from eight different electronic health records that aim to be utilized for detecting a situation where the statistical properties of the clinical data change over time, i.e., concept drift in machine learning. Regarding this, they collected crowd-sourced labels and compared them with the result of experts. Also, they validated their data by employing some evaluation metrics, such as sensitivity, PPV, specificity, and MCC.This article effectively introduces their dataset and follows the required guideline as a data note article. Also, this reviewer believes that this dataset can contribute to building a time-series model in a clinical setting, deriving how to monitor key performance indicators when operating a healthcare service, and even identifying general concept drift points.However, before accepting and publishing this paper, this paper should resolve the following issues.i) As mentioned, there are different state-of-the-art concept drift techniques in the data science discipline. The authors consider comparing the human-labeled results with the machine-labeled results using the concept drift techniques.ii) The dataset only includes the location of the data change as labels. However, we can map data change points into several categories, such as sudden change, blip change, recurring change, etc. Thus, authors need to consider those types of changes to extend the reusability of the data.iii) The authors need to suggest a couple of practical examples for utilizing the dataset in a healthcare setting.iv) All figures have poor quality. They need to be improved before publishing.

Level of Interest

Please indicate how interesting you found the manuscript: Choose an item.

Quality of Written English

Please indicate the quality of language in the manuscript: Choose an item.

Declaration of Competing Interests

Please complete a declaration of competing interests, considering the following questions:

- Have you in the past five years received reimbursements, fees, funding, or salary from an organisation that may in any way gain or lose financially from the publication of this manuscript, either now or in the future?

- Do you hold any stocks or shares in an organisation that may in any way gain or lose financially from the publication of this manuscript, either now or in the future?
- Do you hold or are you currently applying for any patents relating to the content of the manuscript?
- Have you received reimbursements, fees, funding, or salary from an organization that holds or has applied for patents relating to the content of the manuscript?
- Do you have any other financial competing interests?
- Do you have any non-financial competing interests in relation to this paper?

If you can answer no to all of the above, write 'I declare that I have no competing interests' below. If your reply is yes to any, please give details below.

I declare that I have no competing interests.

I agree to the open peer review policy of the journal. I understand that my name will be included on my report to the authors and, if the manuscript is accepted for publication, my named report including any attachments I upload will be posted on the website along with the authors' responses. I agree for my report to be made available under an Open Access Creative Commons CC-BY license (http://creativecommons.org/licenses/by/4.0/). I understand that any comments which I do not wish to be included in my named report can be included as confidential comments to the editors, which will not be published.

Choose an item.

To further support our reviewers, we have joined with Publons, where you can gain additional credit to further highlight your hard work (see: https://publons.com/journal/530/gigascience). On publication of this paper, your review will be automatically added to Publons, you can then choose whether or not to claim your Publons credit. I understand this statement.

Yes Choose an item.
