# Supplementary material for: Health record hiccups—5,526 real-world time series with change points labelled by crowdsourced visual inspection
Source: Gigascience. 2023 Jul 28;12:giad060. doi: 10.1093/gigascience/giad060 (PMC10375518; doi:10.1093/gigascience/giad060)
Supplement: giad060_Reviewer_3_Report_Original_Submission — Feng Xie -- 4/19/2023 Reviewed [file giad060_reviewer_3_report_original_submission.pdf]

Reviewer Report

Title: Health Record Hiccups - 5526 real-world time series with change points labelled by crowd-sourced visual inspection

Version: Original Submission **Date:** 4/19/2023

Reviewer name: Feng Xie

Reviewer Comments to Author:

The study by Quan et al. describes a large collection of labeled EHR time series that can be used to validate automated methods for change point detection. There are 5526 time series generated from 8 different EHR datasets, engaging over 2000 citizen-science volunteers. This study tried to present a large collection of labeled EHR time-series for open and reproducible research. However, there are some concerns in terms of the research gap and implications. Some important information might be missing. I have summarized some major points below.1. I didn't well understand the research gap and unmet need the authors wanted to address. Please address why the change point detection in EHR is important and should be addressed. It would be better to give some real-world examples to let readers have s clear understanding of the importance of your question. The research gap should also be well-identified in the introduction by citing some references. I noticed that few references were cited in the introduction. The authors should add more references to prove that what you present is credible and related to previous research.2. It would be nice to give a figure to show the whole workflow. I feel there are so many technical details in the manuscript, and the author might want to organize these in clear logic to make your workflow easy to understand.3. It would be good to present a more overall descriptive analysis of the data to show us the essential characteristics of the information, especially since this is a data descriptor. We care more about what your data look like instead of some technical details on how you extract data.4. I notice some grammar errors, and please proofread to minimize them. Also, the last sentence of the first paragraph is too long.5. I would suggest adding a substantial paragraph on the implication of your study. It can include how your data can be used in the real world or where future researchers can utilize your data. These should be elaborated on and fully discussed to show that your study/data is of impact.

Level of Interest

Please indicate how interesting you found the manuscript: Choose an item.

Quality of Written English

Please indicate the quality of language in the manuscript: Choose an item.

Declaration of Competing Interests

Please complete a declaration of competing interests, considering the following questions:

- Have you in the past five years received reimbursements, fees, funding, or salary from an organisation that may in any way gain or lose financially from the publication of this manuscript, either now or in the future?
- Do you hold any stocks or shares in an organisation that may in any way gain or lose financially from the publication of this manuscript, either now or in the future?
- Do you hold or are you currently applying for any patents relating to the content of the manuscript?
- Have you received reimbursements, fees, funding, or salary from an organization that holds or has applied for patents relating to the content of the manuscript?
- Do you have any other financial competing interests?
- Do you have any non-financial competing interests in relation to this paper?

If you can answer no to all of the above, write 'I declare that I have no competing interests' below. If your reply is yes to any, please give details below.

I declare that I have no competing interest

I agree to the open peer review policy of the journal. I understand that my name will be included on my report to the authors and, if the manuscript is accepted for publication, my named report including any attachments I upload will be posted on the website along with the authors' responses. I agree for my report to be made available under an Open Access Creative Commons CC-BY license (http://creativecommons.org/licenses/by/4.0/). I understand that any comments which I do not wish to be included in my named report can be included as confidential comments to the editors, which will not be published.

Choose an item.

To further support our reviewers, we have joined with Publons, where you can gain additional credit to further highlight your hard work (see: https://publons.com/journal/530/gigascience). On publication of this paper, your review will be automatically added to Publons, you can then choose whether or not to claim your Publons credit. I understand this statement.

Yes Choose an item.
