# Supplementary material for: Health record hiccups—5,526 real-world time series with change points labelled by crowdsourced visual inspection
Source: Gigascience. 2023 Jul 28;12:giad060. doi: 10.1093/gigascience/giad060 (PMC10375518; doi:10.1093/gigascience/giad060)
Supplement: giad060_Reviewer_3_Report_Revision_1 — Feng Xie -- 6/7/2023 Reviewed [file giad060_reviewer_3_report_revision_1.pdf]

Reviewer Report

Title: Health Record Hiccups - 5526 real-world time series with change points labelled by crowd-sourced visual inspection

Version: Revision 1 **Date:** 6/7/2023

Reviewer name: Feng Xie

Reviewer Comments to Author:

Thank the authors for the revision. The response has addressed my concerns. The overall quality and structure have been improved. The logic is clearer now.One outstanding issue is that I am still not very satisfied with the discussion part. The authors have added more applications to this data and how future researchers can use your data (I feel it's very important as this is a data note, and the main aim is to involve future researchers in using your data). But these are not well elaborated, and the citation/references were not fully provided. For example, when the authors talked about "the primary audience for this dataset is developers of (univariate) change point detection methods", it's better to have some references and citations (what are they?), and talk more about HOW the data can be used, rather than the lack of publicly-available data. Also, when the author mentioned the application of this dataset to AutoML and outlier detection, no references were cited. It's better to cite some papers to show the audience HOW they used the dataset to develop a method or more.

Level of Interest

Please indicate how interesting you found the manuscript: Choose an item.

Quality of Written English

Please indicate the quality of language in the manuscript: Choose an item.

Declaration of Competing Interests

Please complete a declaration of competing interests, considering the following questions:

- Have you in the past five years received reimbursements, fees, funding, or salary from an organisation that may in any way gain or lose financially from the publication of this manuscript, either now or in the future?
- Do you hold any stocks or shares in an organisation that may in any way gain or lose financially from the publication of this manuscript, either now or in the future?
- Do you hold or are you currently applying for any patents relating to the content of the manuscript?
- Have you received reimbursements, fees, funding, or salary from an organization that holds or has applied for patents relating to the content of the manuscript?

- Do you have any other financial competing interests?
- Do you have any non-financial competing interests in relation to this paper?

If you can answer no to all of the above, write 'I declare that I have no competing interests' below. If your reply is yes to any, please give details below.

I declare that I have no competing interests

I agree to the open peer review policy of the journal. I understand that my name will be included on my report to the authors and, if the manuscript is accepted for publication, my named report including any attachments I upload will be posted on the website along with the authors' responses. I agree for my report to be made available under an Open Access Creative Commons CC-BY license (http://creativecommons.org/licenses/by/4.0/). I understand that any comments which I do not wish to be included in my named report can be included as confidential comments to the editors, which will not be published.

Choose an item.

To further support our reviewers, we have joined with Publons, where you can gain additional credit to further highlight your hard work (see: https://publons.com/journal/530/gigascience). On publication of this paper, your review will be automatically added to Publons, you can then choose whether or not to claim your Publons credit. I understand this statement.

Yes Choose an item.
